# Accounting for response styles in rating data using IRT and IRTree models: A tutorial in R with applications to self-reported metacognitive competence

**DOI:** 10.3758/s13428-026-03181-z

**Published:** 2026-10-14

**Authors:** Thorsten Meiser, Esther Ulitzsch, Mirka Henninger

**Affiliations:** 1 School of Social Sciences, Department of Psychology, University of Mannheim, Mannheim, Germany; 2 Centre for Educational Measurement (CEMO) and Centre for Research on Equality in Education (CREATE), University of Oslo, Oslo, Norway; 3 Faculty of Psychology, University of Basel, Basel, Switzerland

**Keywords:** IRT models, IRTree models, Response styles, Generalized partial credit model, Metacognition

## Abstract

Response styles refer to individual differences in the use of the item response format irrespective of item content. They include tendencies to favor or avoid midpoint or extreme response categories, which can distort the measurement of latent constructs. This tutorial provides an overview of IRT and IRTree models that accommodate response style factors, allowing researchers to analyze response-style effects and adjust latent-trait estimates for potential bias. In particular, we give a practical guide to using IRT and IRTree models for response styles and illustrate the workflow of model implementation, estimation, and interpretation with an empirical data set on self-reported metacognitive competence (Double, 2025). Throughout the tutorial, IRT and IRTree models are specified with standard R tools, and parameter estimation is accomplished in the common framework of multidimensional IRT with the R package mirt (Chalmers, 2012; Chalmers et al., 2025). The empirical results demonstrate that IRT and IRTree models can disentangle substantive traits and response styles, and that model-based estimates of substantive traits are adjusted accordingly. More specifically, both model classes reveal pronounced correlations among latent trait dimensions and among response style factors, but only small correlations between traits and response styles. In addition, the model-based trait estimates show the expected patterns of amplification and shrinkage after adjusting for midscale and extreme response style. The tutorial concludes with a discussion of recent developments in psychometric modeling.

## Introduction

The assessment of latent constructs via self-report measures is commonplace in cognitive and social psychology, personality research, and educational studies. Self-report measures predominantly rely on responses to items with a Likert response format, where individuals give gradual ratings of the degree to which a given statement applies to them. Such rating responses are contaminated, however, with individual differences in general response tendencies and therefore do not provide pure reflections of the latent constructs that are to be measured. Among the response tendencies that can affect and bias self-report ratings are social desirability (Paulhus, 1991; Ziegler et al., 2011), careless responding (Huang et al., 2015; Ulitzsch et al., 2022) and response styles (Baumgartner & Steenkamp, 2001; Podsakoff et al., 2012). Response styles refer to individual differences in the interpretation and use of the response categories, which can manifest, for example, in a systematic tendency to prefer or avoid midscale categories (MRS: midscale response style) or extreme categories (ERS: extreme response style) irrespective of the item content. Unlike susceptibility to social desirability and careless responding, which vary as a function of high-stakes versus low-stakes contexts or cognitive burden (e.g., Seitz et al., 2025; Ulitzsch et al., 2024b), response styles such as midscale or extreme responding are intricately connected to the use of rating scale response formats. At the same time, they can bias individual test scores, increase statistical uncertainty in trait estimates, inflate or deflate relationships between measures, and distort group comparisons (Adams et al., 2019; Bolt & Meng, 2025; Moors, 2003; Morren et al., 2012; Ulitzsch et al., 2024a; Van Vaerenbergh & Thomas, 2013). Researchers who use Likert-type scales to measure constructs of interest should therefore be aware of the role of individual differences in response styles and consider tools to account for their potentially deleterious effects on conclusions drawn from self-report measures. This tutorial presents and illustrates such methodological tools for researchers in various fields of psychology.

Several statistical approaches have been proposed to account for response styles in the framework of item response theory (IRT). In a nutshell, IRT models enable researchers to specify the relationship between presumed underlying response processes and observed item responses in a theory-driven way, thereby enabling the measurement of latent constructs disentangled from other influences like MRS and ERS. Prominent IRT approaches to capture response styles were introduced as extensions of traditional IRT models for ordinal item responses with additional person dimensions or with random effects on threshold parameters that determine which response category is the most likely one for a respondent and their trait value (Bolt et al., 2014; Falk & Cai, 2016; Jin & Wang, 2014; Wang & Wu, 2011). Alternatively, multi-process IRT decision tree (IRTree) models have been used to analyze and control response styles in self-report rating data (Böckenholt, 2017; Khorramdel & von Davier, 2014; Meiser et al., 2019; Plieninger & Meiser, 2014). These models decompose observed responses into a series of theoretically presumed decision processes that can include choosing response categories based on MRS and ERS. The different approaches have been discussed in recent theoretical (Bolt & Meng, 2025) and formal (Falk & Ju, 2020; Henninger & Meiser, 2020a) reviews, and they were compared in empirical and simulation-based analyses (Böckenholt & Meiser, 2017; Schoenmakers et al., 2024). Complementary to these theoretically and statistically oriented overviews, the present tutorial provides a practical step-by-step guide to controlling for response styles with extended ordinal IRT and multi-process IRTree models. To this end, we illustrate the workflow for model implementation, estimation, and interpretation using an empirical data example and standard software for psychometric analysis.

The data example is taken from research on self-reported metacognitive competence by Double 2025. In two studies, the author administered a set of eight items from a larger item pool to assess subjective metacognitive ability. The items were divided into two subscales of four items each, labeled “online monitoring” (e.g., “I deliberately assess my performance on all tasks”) and “metacognitive awareness” (e.g., “I’m realistic about my capabilities”). The items had a six-point rating format with categories ranging from 1-“strongly disagree” to 6-“strongly agree”. Participants were recruited from the same population following identical criteria in both studies, so that we merged the item responses for our empirical illustration.

In this tutorial, we show how IRT and IRTree models accounting for response styles can be applied with standard IRT software in the R framework for statistical computing (R Core Team, 2025). More specifically, we chose the R package mirt (Chalmers, 2012; Chalmers et al., 2025) that allows researchers to implement a variety of IRT models and to impose or release parameter restrictions in a user-friendly way. This flexibility enables us to demonstrate straightforward solutions to common challenges in applying multidimensional IRT and IRTree models for response styles, including parameter fixations, equality constraints, overcoming built-in restrictions, and handling data peculiarities such as zero frequencies and empirical or structural missing responses. In the following, we assume that readers are familiar with the general principles of IRT models, and refer to Embretson and Reise 2000 for an introduction.

## Accounting for response styles with extended IRT models for ordinal data

To illustrate how researchers can account for response styles with extended IRT models, we start with the Partial Credit Model (PCM) introduced by Masters 1982 as a prominent psychometric model for ordinal item responses. The PCM specifies the probability of observing a given response category *k* as a function of a person parameter θ and a vector of item-specific threshold parameters $\boldsymbol{\beta }$. For the categories of a six-point rating scale with response categories k=0,…,5, for instance, the probability of response $X_{vi}$ of person *v* to item *i* is defined as

1 $$\begin{aligned} p(X_{vi}=k \mid \theta _v, \boldsymbol{\beta }_i) \; = \; \frac{\displaystyle \exp \left( k \, \theta _v - \sum _{j=0}^k \beta _{ij} \right) }{\displaystyle \sum _{l=0}^5 \exp \left( l \, \theta _v - \sum _{j=0}^l \beta _{ij} \right) } \end{aligned}$$

with $\beta _{i0}:=0$. Following the rationale of the Rasch model for binary item responses (Rasch, 1960), the PCM assumes that all items have the same discrimination with respect to the underlying trait dimension θ. This assumption is released in the generalized Partial Credit Model (gPCM; Muraki, 1992), which includes an item-specific discrimination parameter $\alpha _i$ in the response function:

2 $$\begin{aligned} p(X_{vi} \!=\! k \mid \theta _v, \alpha _i, \boldsymbol{\beta }_i) \! = \! \frac{\displaystyle \exp \left( \alpha _i \left( k \, \theta _v - \sum _{j=0}^k \beta _{ij} \right) \right) }{\displaystyle \sum _{l=0}^5 \exp \left( \alpha _i \left( l \, \theta _v - \sum _{j=0}^l \beta _{ij} \right) \right) } \end{aligned}$$

The PCM and gPCM are divide-by-total models (Thissen & Steinberg, 1986), where the denominator ensures that the category probabilities add up to one for all parameter values, and they form special cases of the (multidimensional) nominal response model (Bock, 1972; Takane & De Leeuw, 1987), which facilitates extensions with additional latent dimensions to capture individual differences in response styles (Henninger & Meiser, 2020a).

**Fig. 1:**
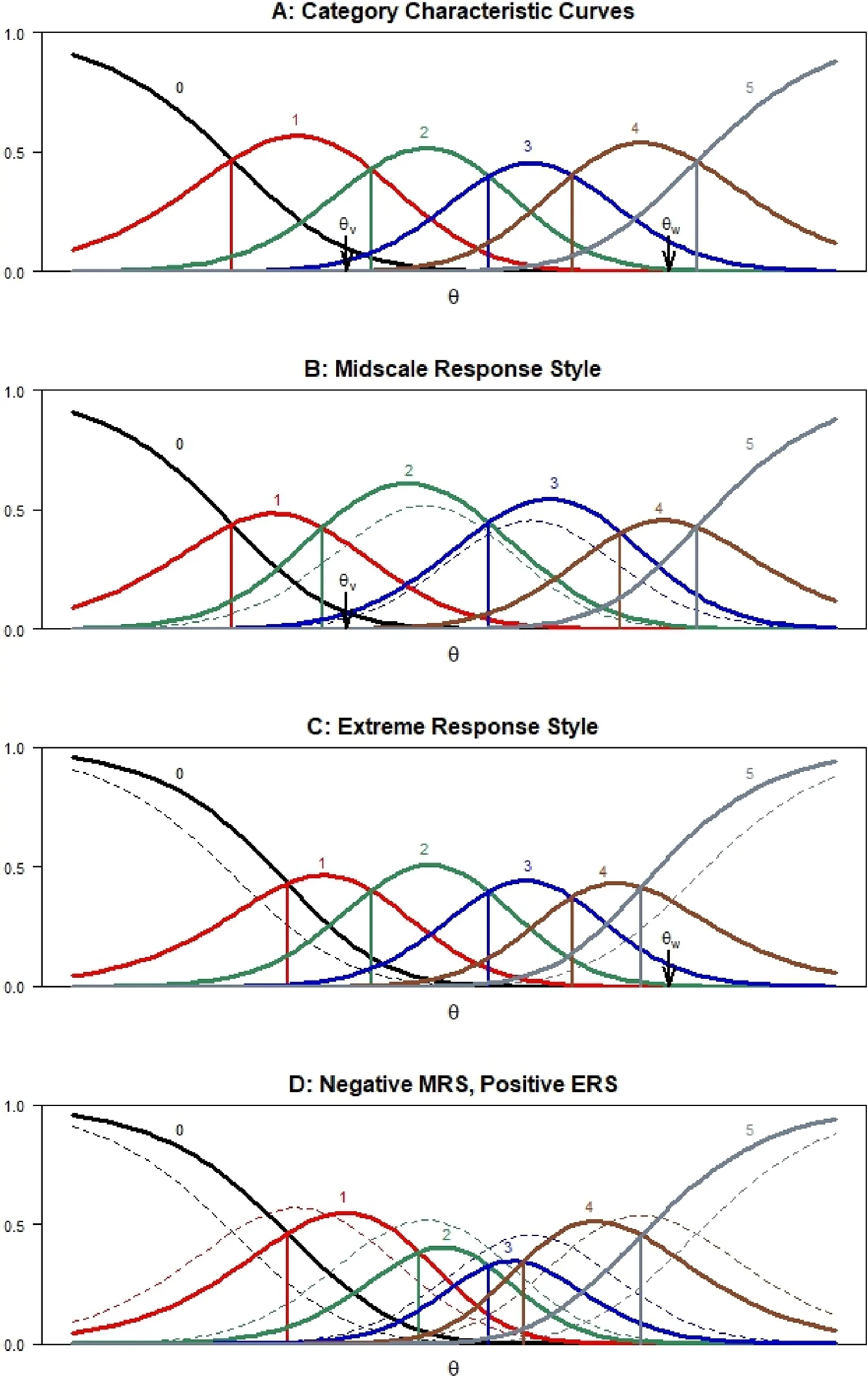
Category characteristic curves in the PCM and gPCM for different kinds of response styles

### Category characteristic curves in the PCM and gPCM

Panel A in Fig. 1 displays the category characteristic curves (CCC) that result from the PCM or gPCM for an item with six response categories. The CCCs depict the probabilities of the response categories k=0,…,5 as a function of the underlying trait θ for a given vector of threshold parameters $\boldsymbol{\beta }_{i}$ and, in the case of the gPCM, additionally for a given discrimination $\alpha _i$. Because the probability of observing the smallest category converges to 1 for $\theta \rightarrow - \infty$ and to 0 for $\theta \rightarrow \infty$, and the probability of observing the largest category converges to 0 for $\theta \rightarrow - \infty$ and to 1 for $\theta \rightarrow \infty$, the CCCs for the two extreme response categories are monotonically decreasing and increasing, respectively. The CCCs for the non-extreme categories, in contrast, are non-monotonic and single-peaked, indicating that increasing values of θ raise the probability of a non-extreme category relative to that of the lower categories, but that the upper categories gain in dominance from some point on the latent θ continuum, which leads to a decline in category probability. Importantly, the threshold parameters $\beta _{ij}$ in Eqs. 1 and 2 reflect the locations of the intersections of adjacent CCCs, that is, the values on the latent continuum where the categories j-1 and *j* are equally likely to be observed (see vertical lines in Fig. 1, panel A). Finally, for a given vector of threshold parameters $\boldsymbol{\beta }_i$, the discrimination parameter $\alpha _i$ of the gPCM determines the steepness of the CCCs, with larger values of $\alpha _i$ yielding more precipitous and contracted CCC trajectories.

As the threshold parameters represent the points of equal category probabilities for adjacent response categories, their spread over the latent trait continuum is indicative of the interpretation and use of the response format: For ordered threshold parameters $\beta _{i(j+1)}>\beta _{ij}$ with j=1,…,4, the interval between $\beta _{ij}$ and $\beta _{i(j+1)}$ defines the section on the latent continuum for which response category *j* attains the modal probability. The threshold distance $\beta _{i(j+1)}-\beta _{ij}$ thus mirrors the “width” of category *j*, that is, the range of θ values for which response category *j* is preferred. Accordingly, the interval from -∞ to the leftmost threshold, and the interval from the rightmost threshold to ∞, define the sections for which the extreme categories 0 and 5 are the most likely responses. The locations of the outer thresholds relative to the other threshold parameters thus reflect the ranges of θ for which the extreme response categories prevail. To illustrate the meaning of threshold parameters and CCCs, panel A of Fig. 1 marks the trait locations $\theta _v$ and $\theta _w$ of two respondents. As can be seen, respondent *v* would most likely choose category 1, whereas respondent *w* would favor category 4.

### Category characteristic curves and response styles

In the PCM and gPCM framework, individual response styles can be conceptualized as increases or decreases in the probabilities of specific response categories above the probabilities predicted from the trait θ alone. Panel B of Fig. 1 displays an MRS tendency, which is characterized by enlarged probabilities of selecting categories 2 and 3 in the two mid CCCs as compared to the probabilities under neutral conditions in panel A (dashed lines in panel B). The heightened CCCs for the two midscale categories align with shifts in threshold locations: the threshold for adjacent categories 1 and 2 shifts left, and the threshold for categories 3 and 4 shifts right. By implication, the two midscale categories show modal probabilities over larger sections of the continuum (compare panels B and A in Fig. 1), and respondent *v* with trait value $\theta _v$ who preferred category 1 under neutral conditions is most likely to select category 2 under MRS.

Likewise, an ERS tendency is characterized by increased probabilities of the extreme categories 0 and 5, as demonstrated by the CCCs in panel C of Fig. 1 (probabilities from panel A as dashed lines). The more pronounced CCCs for the two extreme categories are accompanied by a shift of the leftmost threshold location to the right and a shift of the rightmost threshold location to the left, thereby producing larger sections with modal probabilities for the extreme categories on either side (compare panels C and A in Fig. 1). Person *w* with trait value $\theta _w$ would therefore be most likely to choose category 5 under ERS rather than the preferred category 4 under neutral conditions. While panels B and C in Fig. 1 highlight the roles of MRS and ERS in isolation, the two response styles can co-occur in combination. Panel D illustrates the CCCs for the case of a negative MRS propensity (i.e., an anti-midscale bias) together with a positive ERS tendency relative to the neutral CCCs in panel A (dashed lines).

To account for individual differences in response styles that evoke variations of the CCCs, extended IRT models have been proposed which specify response styles either in terms of additional person dimensions that increase or decrease the probabilities of distinct response categories (e.g., Adams et al., 2019; Bolt and Newton, 2011; Bolt et al., 2014; Falk and Cai, 2016; Wetzel and Carstensen, 2017) or in terms of random effects on the threshold parameters (e.g., Henninger, 2021; Jin and Wang, 2014; Wang and Wu, 2011). As is apparent from our discussion of Fig. 1, however, changes of category probabilities and shifts of threshold parameters are intricately intertwined, so that the different specifications can be considered formally equivalent techniques in an integrative framework of IRT models for response styles (Henninger & Meiser, 2020a). An extended version of the gPCM to accommodate response styles can be written as

3 $$\begin{aligned} p(X_{vi}=k \mid \theta _v, \boldsymbol{\eta }_v, \alpha _i, {\boldsymbol{d}}_i) \; = \; \frac{\displaystyle \exp \left( \alpha _i \, k \, \theta _v \, + \, \sum _{j=0}^k s_j \, \eta _{jv} \, + \, d_{ik} \right) }{\displaystyle \sum _{l=0}^5 \exp \left( \alpha _i \, l \, \theta _v \, + \, \sum _{j=0}^l s_j \, \eta _{jv} \, + \, d_{il} \right) } \end{aligned}$$

with $d_{i0}:=0$. The new vector of category-specific factors $\boldsymbol{\eta }_{v}$ captures the values of respondent *v* on the presumed response style dimensions for the categories k=0,…,5. The dimensions are weighted by a priori determined scoring weights $s_j$ for response category *j* and response style $\eta _j$ (e.g., Falk & Ju, 2020). To introduce an MRS dimension, we can include a joint dimension for the midscale response categories 2 and 3, $\eta _2=\eta _3=\eta _{\text {MRS}}$, with scoring weight vector (0, 0, 1, 1, 0, 0). Thereby, a positive value of $\eta _{\text {MRS}}$ leads to an increase in the probabilities for midscale category responses (see Fig. 1, panel B), whereas negative values lead to a decrease. In the same vein, we can include a joint ERS dimension for the extreme categories 0 and 5, $\eta _0=\eta _5=\eta _{\text {ERS}}$, with scoring weight vector (1, 0, 0, 0, 0, 1). Positive values of $\eta _{\text {ERS}}$ thus result in heightened response probabilities for the two extreme categories (see Fig. 1, panel C), and negative values result in lowered response probabilities.

Comparing Eq. (2) and its multidimensional extension in Eq. 3, two comments are in place. First, the cumulating threshold parameters $\sum _{j=0}^k \beta _{ij}$ and their negative sign in Eq. 2 are replaced with intercept parameters $d_{ik}$ and a positive sign in Eq. 3. This change of notation is made to achieve consistency with the following mirt analyses. The R package mirt allows researchers to obtain estimates of the threshold parameters $\beta _{ij}$ if the item response function includes only one latent person dimension, but it generates output for estimated intercept parameters $d_{ik}$ in the case of within-item multidimensionality.^1^ It can easily be seen, however, that the gPCM in Eq. 2 follows as a special case of Eq. 3 with $d_{ik} = - \alpha _i \sum _{j=0}^k \beta _{ij}$ if no additional dimensions $\eta _j$ are specified. Second, while the discrimination or loading with respect to the trait dimension θ is specified to be item-specific with parameter $\alpha _i$, loadings on the response style dimensions $\eta _j$ are kept constant across items in Eq. 3. This constraint reflects the assumption that the strength of response style effects is independent of item content, so that the items do not vary in their sensitivity to individual differences in MRS and ERS. However, the constraint can be easily relaxed if theoretical propositions suggest item-specific sensitivity to response styles (Falk & Cai, 2016; Henninger & Meiser, 2020b).

Because response style analyses are often conducted in the context of multidimensional constructs, like distinct subfactors of a common latent variable (Böckenholt, 2017) or the Big Five personality dimensions (Henninger & Meiser, 2020b; von Davier & Khorramdel, 2013), we can finally generalize Eq. 3 to incorporate a person vector $\boldsymbol{\theta } = (\theta _1,\ldots ,\theta _H)$ of multiple substantive traits:

4 $$\begin{aligned} p(X_{vi} \!=\! k \mid \boldsymbol{\theta }_v, \boldsymbol{\eta }_v, \boldsymbol{\alpha }_i, {\boldsymbol{d}}_i) \! = \! \frac{\displaystyle \exp \left( k \, \sum _{h=1}^H \alpha _{hi} \, \theta _{hv} \! + \! \sum _{j=0}^k s_j \, \eta _{jv} \! + \! d_{ik} \right) }{\displaystyle \sum _{l=0}^5 \exp \left( l \, \sum _{h=1}^H \alpha _{hi} \, \theta _{hv} \! + \! \sum _{j=0}^l s_j \, \eta _{jv} \! + \! d_{il} \right) } \end{aligned}$$

Here we assume that each item refers to one of the latent trait dimensions $\theta _h$, corresponding to a simple structure model in confirmatory factor analysis, such that for each item *i* only one loading parameter $\alpha _{hi}$ is estimated and all other loading parameters are a priori fixed to zero. Since response styles are assumed to be independent of item content, the number of substantive trait dimensions does not alter the dimensionality or structure of the modeled response style factors. The generalization in Eq. 4 allows researchers to account for response styles across different latent trait dimensions and will thus be used in the following section for the analysis of self-reported metacognitive competence with the two subfactors of “online monitoring” and “metacognitive awareness” (Double, 2025).

### Modeling response styles in self-reported metacognitive competence

We illustrate the use of ordinal IRT models for response styles with the eight six-point rating items on self-reported metacognitive competence presented by Double 2025 and with the R package mirt (Chalmers, 2012; Chalmers et al., 2025). Although the categories of the six-point rating scale were coded 1,…,6 in the original data, mirt automatically treats the lowest observed category as category k=0 according to Eqs. 1 to 4, so that no recoding is required for the analyses. We merged the data from Study 1 and Study 2 in Double 2025 and applied the same attention screening criteria that were used in the original studies. Moreover, we discarded the data of one respondent with missing values in all eight metacognitive items. The procedure of data preparation led to an effective sample size of n=477 respondents.^2^ For the following psychometric analyses, the response data from the two studies were stored in a joint data.frame named joint.data. The R script with code for data preparation and for the analyses in this tutorial plus some additional analyses can be retrieved from https://osf.io/cbwun/files/osfstorage.

As a first screening step, we recommend that researchers inspect item response frequencies to see whether responses are generally skewed toward the lower or higher categories and to detect potential zero-category frequencies. Skewed response frequencies may signal that the items are relatively easy or relatively difficult for the sample of respondents, which can affect the interpretation of response style factors (Merhof et al., 2024; Schoenmakers et al., 2024). Zero category frequencies have to be taken into account when interpreting the threshold or intercept parameters from the mirt output and for the specification of scoring weight vectors in multidimensional models (see below). For the eight items on metacognitive competence, the item response frequencies can be displayed as follows:

**Figure:**
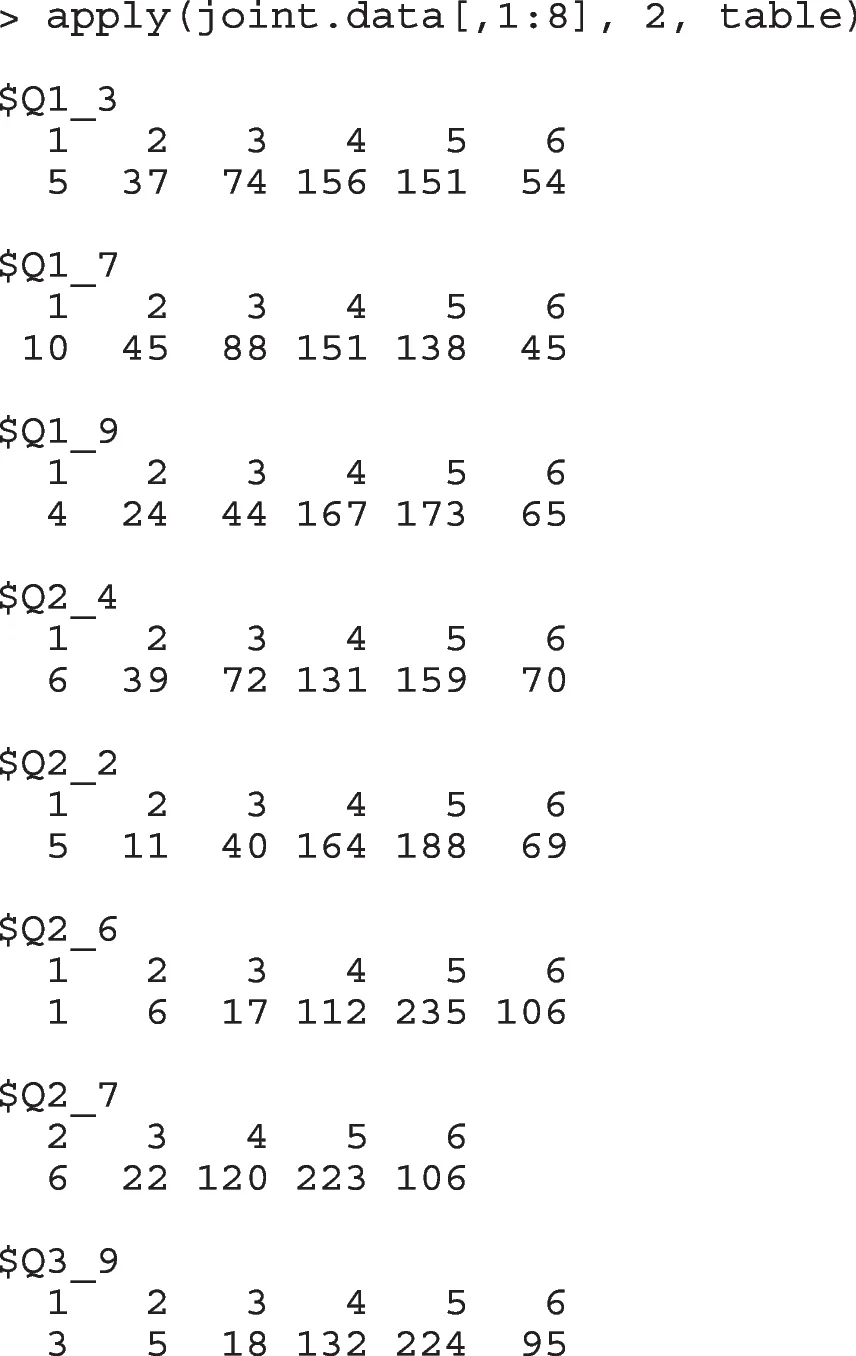

The frequencies show a general tendency towards the higher categories. That is, overall, respondents ascribe high levels of metacognitive competence to themselves, reflecting the relative ease of the items in a technical sense. At the same time, the seventh item shows a zero frequency for the first response category, as only the response categories 2,…,6 were observed for this item. Both observations will be relevant in the following analyses.

#### Baseline models without response styles

We start with the PCM and gPCM without response styles in Eqs. 1 and 2 as baseline models. Because the items on metacognitive competence comprise two subscales, with the first four items referring to the latent dimension ”online monitoring” and the second four items referring to the latent dimension ”metacognitive awareness”, we need to define the model structure with the function mirt.model:

**Figure:**
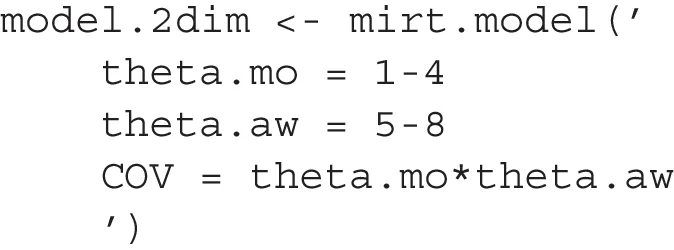

The resulting object model.2dim specifies two latent trait variables θ, labeled theta.mo for “online monitoring” and theta.aw for “metacognitive awareness’, with a full variance covariance matrix COV. Although the overall model structure is two-dimensional, it maintains within-item unidimensionality by defining unidimensional item response functions for each subscale: items 1–4 and items 5–8. The joint PCM for the two subscales of metacognitive competence can be run by selecting the mirt.model model.2dim and by choosing itemtype=’Rasch’ in the mirt command:

**Figure:**
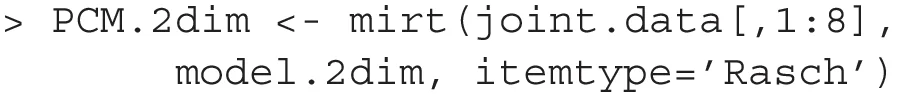

Likewise, a joint gPCM for the two subscales can be estimated by setting itemtype=’gpcm’:

**Figure:**
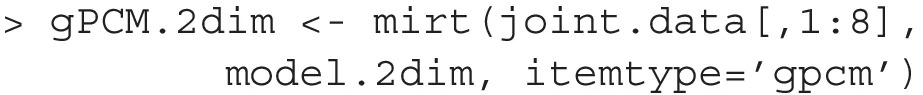

As the PCM is a special case of the gPCM, the two models can be compared with a likelihood-ratio test using the command anova:

**Figure:**
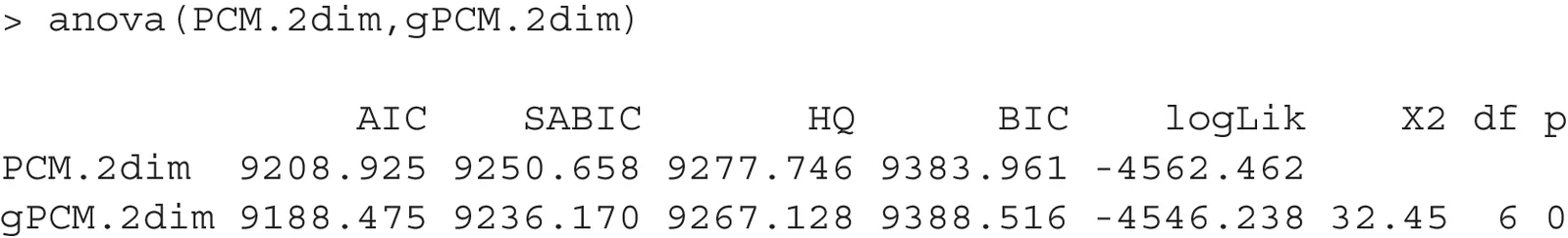

The significant result suggests that the more restrictive PCM provides a poorer fit to the empirical responses than the superordinate gPCM with item-specific discrimination parameters. Based on the information criteria AIC and BIC for model selection, AIC indicates a better compromise between model fit and parsimony for the gPCM, whereas the lower BIC value favors the PCM.

For the following analyses, we select the more complex gPCM as the baseline model because it shows superior fit over the PCM according to the likelihood-ratio test and because it allows us to demonstrate the more general case. The parameter estimates can be obtained in a tabular format and in the threshold notation of Eq. 2 with the options simplify=TRUE and IRTpars=TRUE in the coef command:

**Figure:**
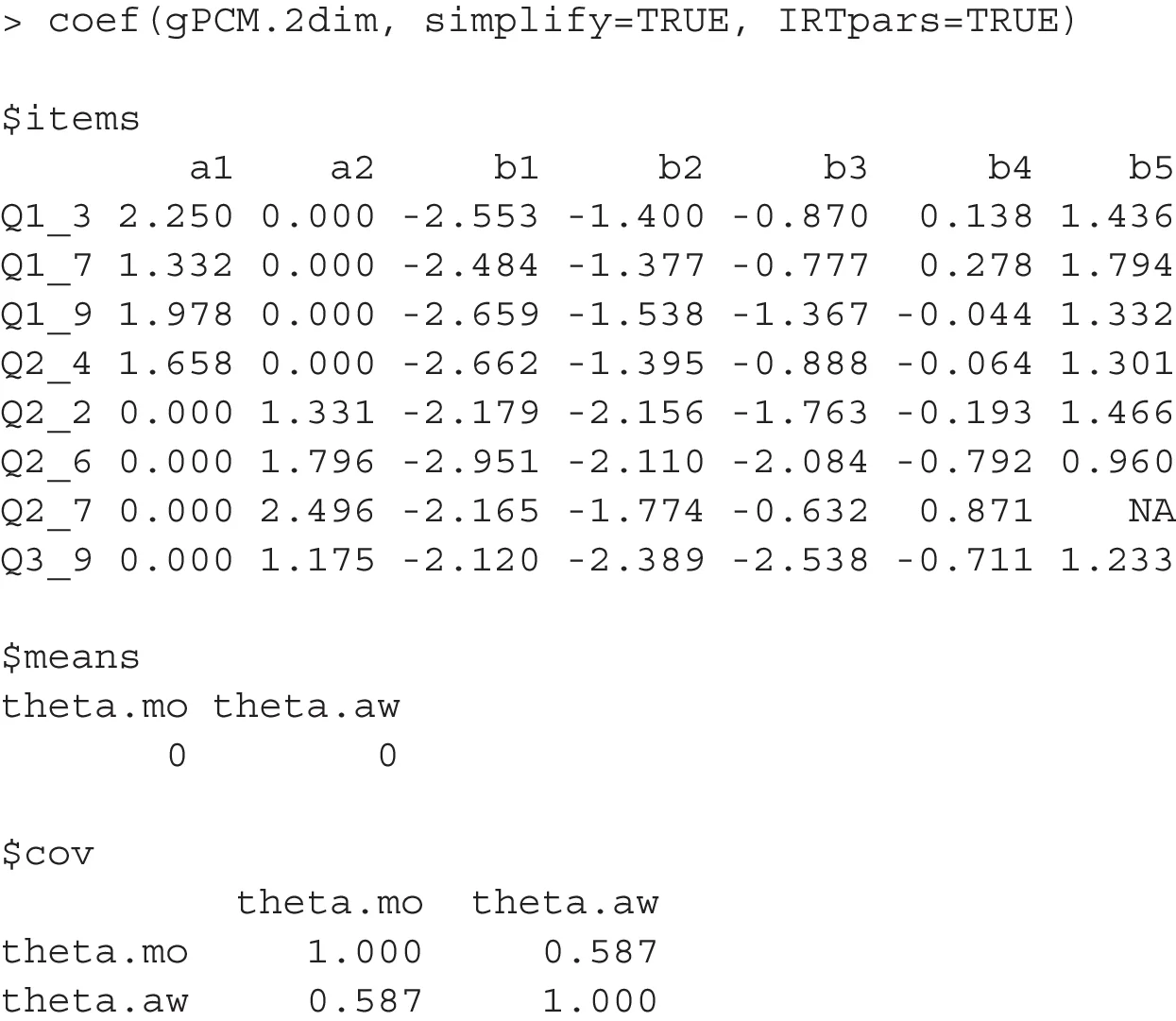

The columns a1 and a2 of the output contain the item-specific discrimination parameters $\alpha _i$ for the two trait dimensions and eight items. The estimated values and the fixed 0.0 values reflect the assignment of items to latent dimensions in the given model structure. The columns b1 to b5 display the threshold parameters $\beta _{i1} { to}\beta _{i5}$ for each item. Except for the last item, the estimated threshold parameters are monotonically ordered, in line with the interpretation of the CCCs as yielding sections of modal category probabilities (see Fig. 1).

The seventh item has a missing threshold, because only the five response categories 2,…,6 were empirically observed for this item, while category 1 had zero frequency (see above). Since the lowest category is equated with the observed category 2 in this situation, four threshold parameters are estimated and listed in columns b1 to b4. The remaining parameters in the output refer to the distribution of the two trait dimensions, which is specified as multivariate normal with expectation vector ${\mu } =(0,0)' ({ i}.{ e}.,\,{\$}$ means in the output) and variance covariance matrix ${\Sigma } ({ i}.{ e}.,\,\$$ cov in the output), where $\sigma ^{2}_{11} { and}\sigma ^{2}_{22}$ are fixed at 1 by default for itemtype=gpcm so that all $\alpha _i$ can be estimated as free parameters.

#### Extended models with response styles

To accommodate individual differences in response styles, we can extend the gPCM with additional latent person dimensions as shown in Eqs. 3 and 4, where an MRS dimension $\eta _{\text {MRS}} { canbeintroducedbydefiningthescoringweightvector}(0,0,1,1,0,0){ forthesixresponsecategories},\,{ andanERSdimension}\eta _{\text {ERS}} { canbespecifiedwithscoringweightvector}(1,0,0,0,0,1).{ Thescoringweightvectorsofthesubstantivelatenttraits}\theta$ follow from Eqs. 1 to 4 as (0,1,2,3,4,5). To implement an extended gPCM including the two traits of metacognitive monitoring and metacognitive awareness and the two additional response style factors of MRS and ERS, we can define the scoring weight matrices concerning the four latent dimensions and six response categories for the eight items as:

**Figure:**
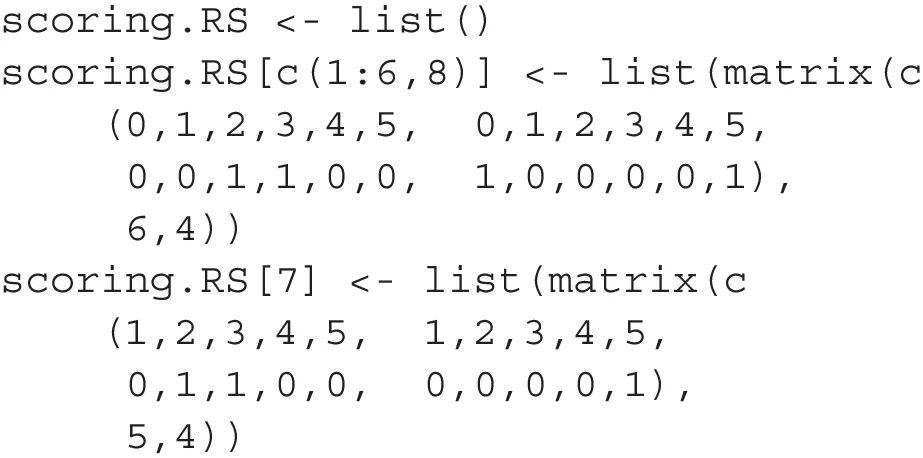

This yields the following scoring weight matrix for the response categories (rows) and dimensions (columns) for items one to six and item eight:

**Figure:**
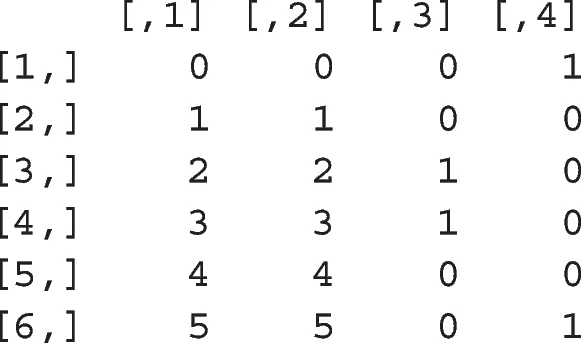

Because only five response categories were observed for the seventh item due to the zero frequency in the lowest category, the scoring weights scoring.RS[7] are tailored accordingly to cover the empirically observed categories for this item, yielding the scoring weight matrix:

**Figure:**
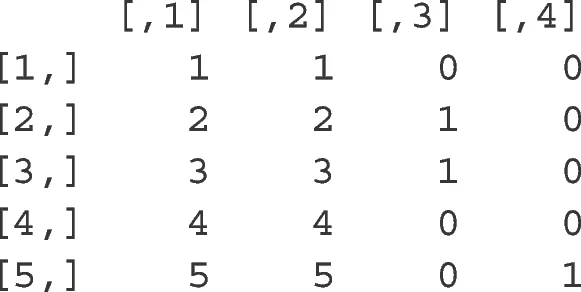

Building on the previous PCM and gPCM analyses, the mirt.model function is employed to expand the model structure to the four latent dimensions:

**Figure:**
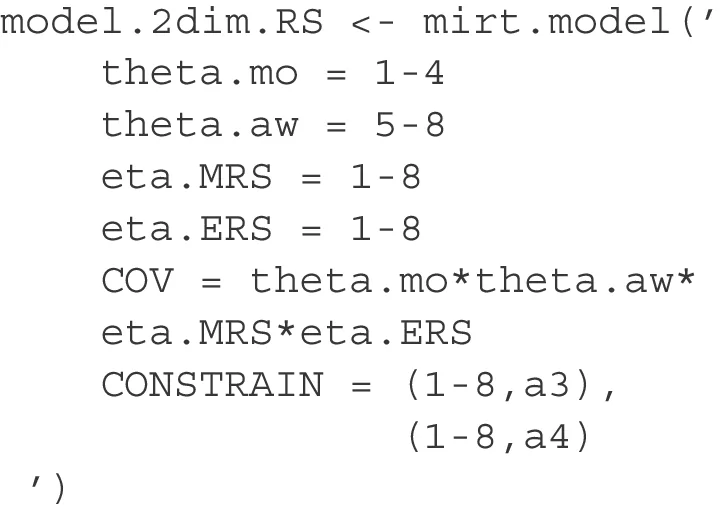

The new mirt.model object model.2dim.RS defines item response functions for the eight items that encompass one substantive trait θ for each item (i.e., metacognitive monitoring or awareness) together with the two response style dimensions ${\eta _\text {MRS}} { and}{\eta _\text {ERS}}$, thus modeling within-item multidimensionality. A full variance–covariance matrix for the four latent dimensions is included with the COV argument. Moreover, the CONSTRAIN command is used to impose equality constraints on the discrimination parameters for the two response style dimensions across items. While these constraints are reflected by omitting discrimination parameters for the $\eta _j$ dimensions in Eqs. 3 and 4, here we specify one freely estimated discrimination parameter per response style dimension because the latent variances are fixed to 1 in the mirt implementation of the gPCM. The implicit restriction of constant discrimination parameters to 1 is thus replaced with a restriction on the latent variances.

The extended gPCM with MRS and ERS dimensions can be estimated by including the scoring weight matrices in the mirt command with the option gpcm_mats=scoring.RS and by using the expanded model structure model.2dim.RS:

**Figure:**
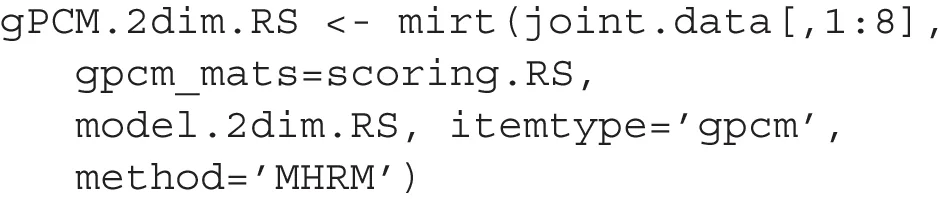

By means of the option method=’MHRM’, we select the Metropolis–Hastings Robbins–Monro algorithm for model estimation (Cai, 2010) that is implemented in mirt and recommended for models with more than two latent dimensions. A model comparison of the extended gPCM accounting for response styles with the baseline gPCM without response style dimensions demonstrates a superior model fit of the extended model:^3^

**Figure:**
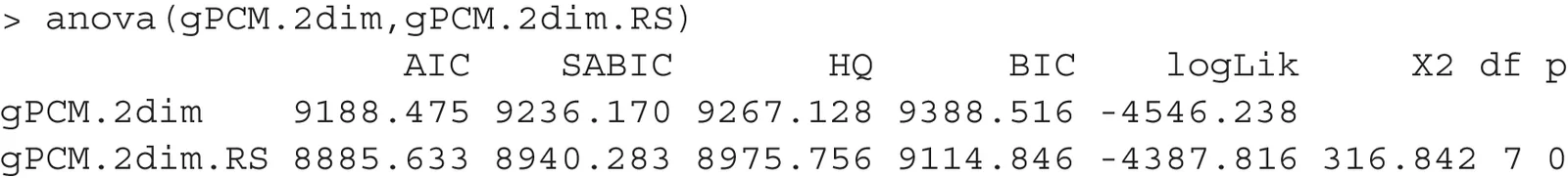

We can therefore conclude that individual differences in response styles affect the observed response behavior over and above the two substantive traits of metacognitive competence.

With the coef command, we can display the estimated factor loadings, the scoring weights that were used in the analysis, the estimated intercept parameters according to Eq. 4, and the parameters for the multivariate distribution of the latent dimensions. Here we show the results for the factor loadings, the intercepts, and the latent correlations of the extended gPCM:

**Figure:**
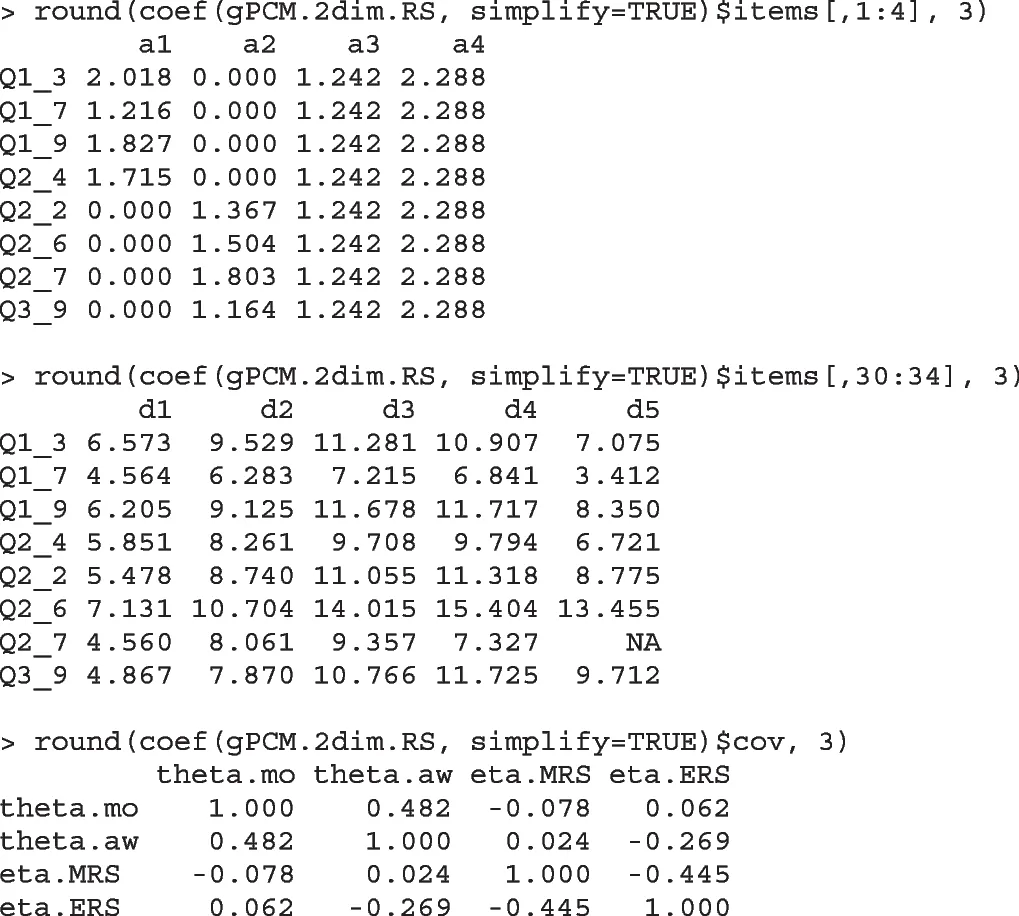

In line with the model specification, the loading parameters on the two substantive dimensions of metacognitive competence were estimated as free parameters for one dimension and fixed to zero for the other dimension (i.e., columns a1 and a2). In contrast, the loading parameters on the two response style dimensions were constrained to be equal across items (i.e., columns a3 and a4). Replicating previous findings (e.g., Böckenholt and Meiser, 2017; Henninger and Meiser, 2020b), ERS has a stronger effect than MRS as evidenced by the larger loading parameters in column a4 than in column a3. Looking at the intercept parameters (i.e., columns d1 to d5), four rather than five parameters were estimated for the seventh item for which only five response categories were observed, paralleling the threshold parameters in our previous analysis of the gPCM. Note, however, that the values of the intercept parameters in the extended gPCM cannot be directly compared with the threshold parameters in the gPCM. As explained above, in contrast to threshold parameters $\beta _{ij}$, the intercept parameters $d_{ik}$ are opposite in sign, weighted by the loading parameters and cumulated over response categories (i.e., $d_{ik} = - \alpha _i \sum _{j=0}^k \beta _{ij}$). Researchers should thus be aware of the different definitions and interpretations of intercept versus threshold parameters.

Of particular interest in the extended gPCM is the estimated variance covariance matrix that reveals the linear relationships between the four dimensions: As the latent variances are fixed to 1, the covariances can be interpreted as correlations and reveal a strong positive relation between the dimensions of metacognitive monitoring (theta.mo) and metacognitive awareness (theta.aw), a strong negative relation between the tendency towards midscale responses (eta.MRS) and the tendency towards extreme responses (eta.ERS), but noticeably weaker relations of the two substantive dimensions of metacognitive competence with the two response style dimensions. This pattern of results supports the convergent validity of the two dimensions of metacognitive competence and the two response style dimensions, while also reflecting discriminant validity between the substantive and response style dimensions. We further note that although the two response style factors exhibit a strong correlation, it is far from a perfect linear dependency, indicating that MRS and ERS represent distinct response style tendencies.

Alternative versions of the extended gPCM can easily be specified by modifying the model structure in the mirt.model function. Two modifications are outlined here for illustration: First, a restricted model variant can be implemented that enforces orthogonality between the two substantive dimensions of metacognitive competence and the two response style dimensions in the latent covariance matrix by adapting the COV line:

**Figure:**
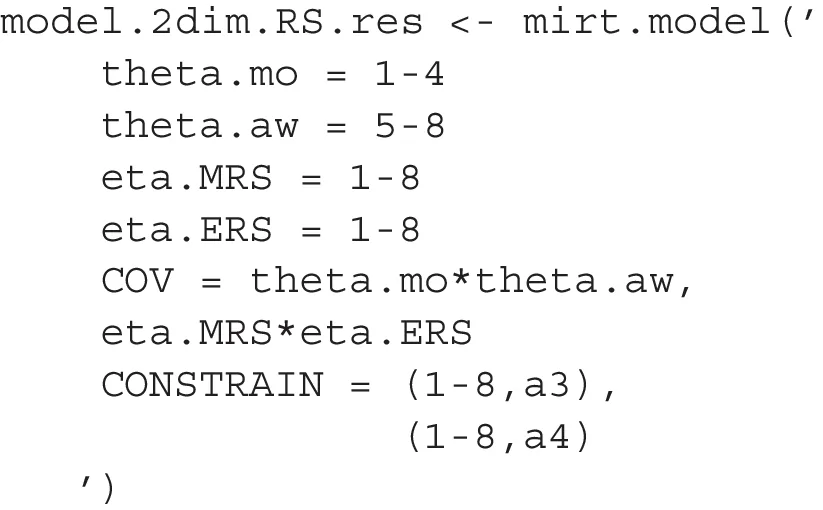

The restricted model bears the assumption that the response style dimensions are uncorrelated with the substantive dimensions of metacognitive competence in the population. Although the full latent variance covariance matrix with linear relations between substantive traits and response style factors can be estimated in models with a priori specified response styles (see Falk and Cai, 2016; Henninger and Meiser, 2020a), it may be worthwhile for theoretical reasons to test such relations by comparing the extended gPCM with full variance covariance matrix and the restricted model version.

Second, the premise of equal loading parameters across items for the two response style dimensions can be relaxed by abandoning the CONSTRAIN command in the model structure model.2dim.RS. The resulting model version thereby allows for item-specific sensitivity to response style effects. The full syntax to implement and estimate these alternative model versions is provided in the R code at https://osf.io/cbwun/files/osfstorage.

#### Adjusting person estimates for response style effects

A major objective of extended IRT models with response style dimensions is to adjust person estimates on the substantive trait dimensions for potential biases due to confounding response styles. For respondents with a high level of MRS, trait values can be expected to be biased toward the center of the trait distribution, that is, low trait values should be overestimated, and large trait values should be underestimated if MRS is not controlled for. Respondents with a low level of MRS, indicating an anti-midscale response tendency, should show the reversed pattern, with trait estimates being shifted toward the extremes, so that low trait values are underestimated and large trait values are overestimated unless MRS is accounted for.

**Fig. 2:**
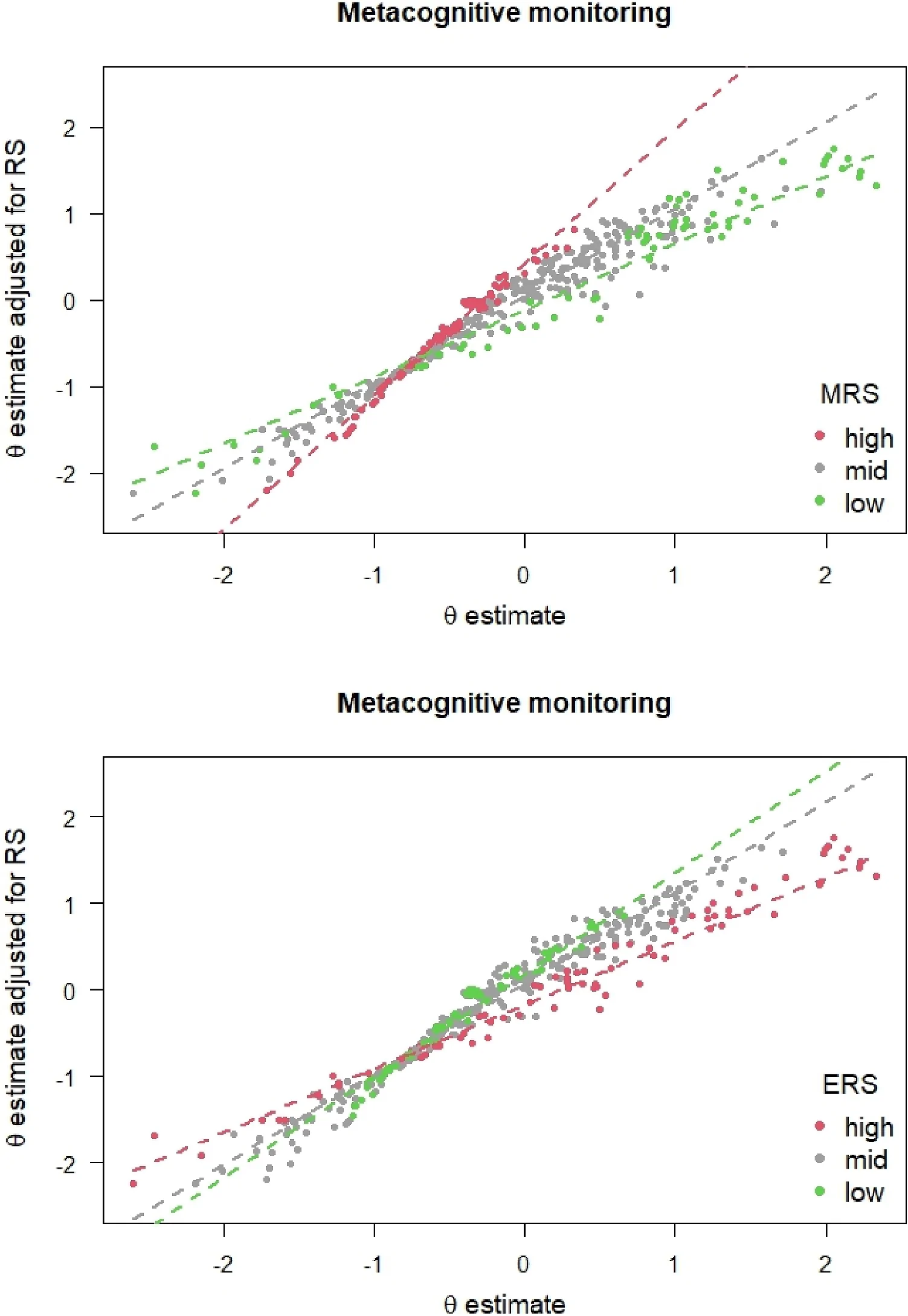
Trait estimates of metacognitive monitoring in the gPCM (*horizontal axis*) and in the extended gPCM (*vertical axis*) for different levels of MRS and ERS

Looking at the given items on metacognitive competence with skewed response frequencies towards higher categories, these mechanisms should especially lead to an underestimation of trait values for respondents with a strong MRS tendency. Extended IRT models for response styles should counteract these biases by amplifying the trait estimates in either direction for high levels of MRS and by shrinking the trait estimates towards the mean for low levels of MRS. This model-implied adjustment effect is illustrated in the upper panel of Fig. 2. The figure shows the trait estimates for the dimension of metacognitive monitoring in the extended gPCM accounting for response styles (vertical axis) as a function of the trait estimates in the baseline gPCM (horizontal axis). Trait estimates were computed using the “expected a posteriori” (EAP) method (see Embretson and Reise, 2000, chapter 7) in both models, which is the default in mirt. The trait estimates are displayed for the upper 20% percentile of MRS (“high”), the middle 60% of the MRS distribution (“mid”), and the lower 20% percentile of MRS (“low”). As predicted, the extended gPCM amplifies the trait estimates in either direction for high levels of MRS, whereas it shrinks the trait estimates towards the mean for low levels of MRS. Due to the skewness of the empirical item responses, which reflect that the items are relatively easy for the sample of respondents, the adjustment is especially evident for the underestimation of positive trait values in combination with high MRS (see red dots and regression line in the upper panel of Fig. 2).

Turning to ERS, high levels of ERS should be associated with an underestimation of lower trait values and an overestimation of larger trait values. In contrast, low values of ERS reflecting an anti-extreme response tendency should coincide with an overestimation of lower trait values and an underestimation of larger trait values. These biases can be accounted for with an extended IRT model that accommodates response styles, as illustrated in the lower panel of Fig. 2: For respondents in the upper 20% percentile of ERS (“high”), the trait estimates in the extended gPCM are shrunk towards the mean relative to the estimates in the baseline gPCM and are, thus, adjusted for the effects of extremity bias. For respondents in the lower 20% percentile of ERS (”low”), in contrast, the estimates in the extended gPCM are amplified in both directions, which reflects the adjustment for anti-extremity bias relative to the baseline gPCM. In line with the skewed response frequencies and relative item easiness, the model-implied adjustment for ERS is particularly pronounced for the overestimation of positive trait values under high ERS (see red dots and regression line in the lower panel of Fig. 2).

## Accounting for response styles with multiprocess IRTree models

Multiprocess IRTree models form a general class of psychometric models that allow researchers to specify and test theoretical assumptions about latent processes underlying observed responses (Böckenholt, 2012; De Boeck & Partchev, 2012; Jeon & De Boeck, 2016). At the core of an IRTree model, observed responses are decomposed into a series of logically contingent judgments or “nodes” that jointly lead to a manifest outcome. The nodes reflect theoretically presumed response processes in terms of binary or ordinal decisions. For each node, an IRT model is specified to parameterize the probabilities of the decision categories. IRTree models have been proposed, for instance, to analyze latent processes underlying different kinds of missing responses in cognitive tests (Debeer et al., 2017), to differentiate latent processes for slow versus fast responses to intelligence items (DiTrapani et al., 2016), and to compare ability estimates and task characteristics as a function of answer change following an initial item response (Jeon et al., 2017). In addition, several psychometric models that have originally been proposed without reference to IRTrees can be subsumed under the umbrella of IRTree models, such as sequential IRT models for repeated item attempts after failure (Culpepper, 2014), dynamic IRT models incorporating response dependencies between items (Andrich et al., 2012; Verhelst & Glas, 1993) or consecutive steps models for ordinal response data (Tutz, 1997; Verhelst et al., 1997). As a general framework, IRTree models disentangle latent response processes over a series of subsequent decision nodes by means of the person and item parameters of node-specific IRT models. Whether the series of nodes implies a temporal sequence of response stages, or merely mirrors logical dependencies, hinges on the data-generating design, such as temporarily ordered processes in designs with answer change (Jeon et al., 2017) as opposed to conditional judgments without temporal order in steps models for singular rating responses (Tutz, 1997; Verhelst et al., 1997).

### IRTree models for response styles

A common field of application for IRTree models has been the analysis of response styles in ordinal rating responses. To account for midscale and extreme response tendencies, some of these models decompose the original responses into a first node separating responses that fall into the midscale categories and responses that provide a clearcut level of agreement or disagreement, followed by subsequent nodes indicating agreement versus disagreement and the choice of an extreme versus non-extreme category (e.g., Böckenholt and Meiser, 2017; Jeon and De Boeck, 2019; Khorramdel and von Davier, 2014; Plieninger and Meiser, 2014). Response processes based on the substantive traits and on MRS and ERS are then represented as latent person dimensions in the node-specific IRT models. Alternative IRTree specifications assign the decision to generally agree versus disagree with the item to the top node of the tree, whereas the choice of moderate categories at the midscale as opposed to extreme categories of agreement or disagreement is modeled in subsequent nodes (e.g., Böckenholt, 2017; Debelak et al., 2025; Meiser et al., 2019). Because choices of agreement and disagreement categories of moderate or extreme intensity do not imply a temporal sequence of the involved processes, there is no natural order in the setup of the IRTrees and the interpretation of the decision nodes solely depends on their definition conditional on superordinate decisions.

**Fig. 3:**
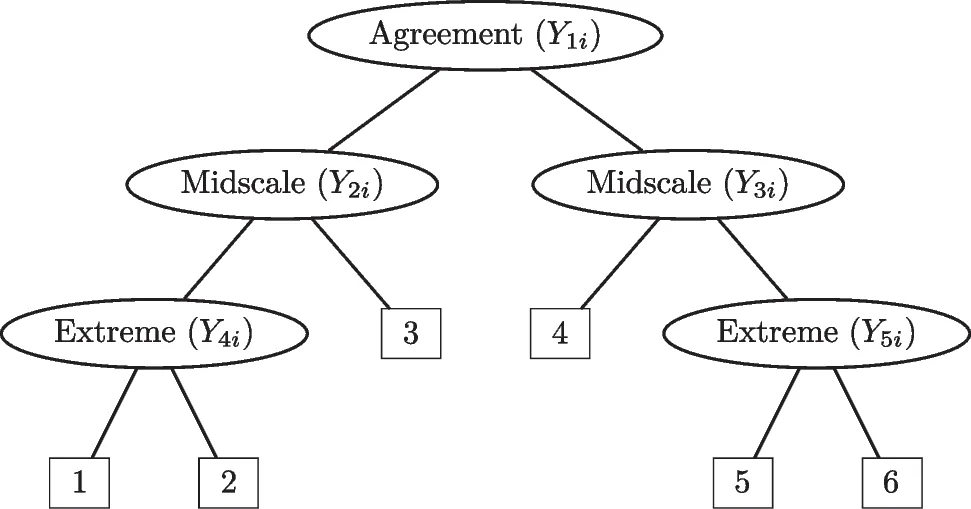
IRTree model for six-point rating responses (adapted from Meiser et al., 2019)

**Table 1:** Coding of the pseudo-items with node-specific IRT models

	Rating category *k*						
	1	2	3	4	5	6	$p(Y_{vgi}=1)$
$Y_{1i}$ (Agreement)	0	0	0	1	1	1	$\frac{\displaystyle \exp (\alpha ^{\text{ a }}_i \theta _{v}+d_{1i})}{\displaystyle 1+\exp (\alpha ^{\text{ a }}_i \theta _{v}+d_{1i})}$
$Y_{2i}$ (Midscale | disagr.)	0	0	1	–	–	–	$\frac{\displaystyle \exp (\alpha ^{\text{ m }}_i \theta _v + \eta _{\text {MRS}v} + d_{2i})}{\displaystyle 1+\exp (\alpha ^{\text{ m }}_i \theta _v + \eta _{\text {MRS}v} + d_{2i})}$
$Y_{3i}$ (Midscale | agr.)	–	–	–	0	1	1	$\frac{\displaystyle \exp (\alpha ^{\text{ m }}_i \theta _v - \eta _{\text {MRS}v} + d_{3i})}{\displaystyle 1+ \exp (\alpha ^{\text{ m }}_i \theta _v - \eta _{\text {MRS}v} + d_{3i})}$
$Y_{4i}$ (Extreme | disagr.)	0	1	–	–	–	–	$\frac{\displaystyle \exp (\alpha ^{\text{ e }}_i \theta _v - \eta _{\text {ERS}v} + d_{4i})}{\displaystyle 1+\exp (\alpha ^{\text{ e }}_i \theta _v - \eta _{\text {ERS}v} + d_{4i})}$
$Y_{5i}$ (Extreme | agr.)	–	–	–	–	0	1	$\frac{\displaystyle \exp (\alpha ^{\text{ e }}_i \theta _v + \eta _{\text {ERS}v} + d_{5i})}{\displaystyle 1+\exp (\alpha ^{\text{ e }}_i \theta _v + \eta _{\text {ERS}v} + d_{5i})}$

Figure 3 displays the IRTree model that will be used herein for the analysis of response styles in the data on self-reported metacognitive competence. The IRTree structure is adapted from previous research (Meiser et al., 2019; see also Böckenholt, 2017; Debelak et al., 2025) and distinguishes five decision nodes for the rating items with response categories k=1,…,6 in Double (2025). The first node captures general disagreement versus agreement with the item content and distinguishes disagreement categories 1, 2, 3 (i.e., the left-branch endpoints) from agreement categories 4, 5, 6 (i.e., the right-branch endpoints). The following two nodes represent choices of the midscale categories 3 or 4 as opposed to non-midscale categories 1, 2 or 5, 6 conditional on disagreement or agreement in the first node. The final two nodes separate extreme responses 1 and 6 from non-extreme responses 2 and 5 conditional on non-midscale decisions of disagreement or agreement in the preceding nodes.

To implement the theoretical IRTree structure in a psychometric framework, the observed responses to each rating item *i* are decomposed into five binary pseudo-items $Y_{1i},\ldots ,Y_{5i}$ that correspond to the nodes of the IRTree (see Fig. 3) and are parameterized through an appropriate IRT model. Table 1 shows the coding of the five pseudo-items together with the node-specific IRT models that capture the presumed influences of the substantive traits and of MRS and ERS in the response process. Because it is assumed that (non-)midscale responses and (non-)extreme responses of disagreement and agreement are jointly driven by the substantive trait and by response styles, we consistently coded all pseudo-items in Table 1 in the direction of increasing response categories, such that larger values of the substantive trait are associated with higher probabilities of outcome 1 (cf. Schoenmakers et al., 2024). As explained in detail below, effects of MRS and ERS are also included as factors in the IRT models for the pseudo-items $Y_{2i}$ and $Y_{3i}$ and for the pseudo-items $Y_{4i}$ and $Y_{5i}$, respectively, according to the direction of coding.

Note that most earlier IRTree applications were limited to unidimensional IRT models for individual nodes, where (non-)midscale and (non-)extreme responses are driven solely by response styles, while the content trait only governs respondents’ decisions of general disagreement versus agreement with item content (e.g., Böckenholt & Meiser, 2017; Jeon & De Boeck, 2019; Plieninger & Meiser, 2014; but see Schoenmakers et al., 2024; von Davier and Khorramdel, 2013). However, such unidimensional parameterizations of IRTree nodes for midscale and extreme responses are prone to conflating trait effects and response style effects (Meiser et al., 2019; Merhof et al., 2024). The simultaneous modeling of trait and response style effects through multidimensional IRT models adopted herein, in contrast, allows us to disentangle substantive traits and response styles in the midscale and extreme nodes of Fig. 3.

Turning to the exact coding and parameterization of pseudo-items for our model analysis in Table 1, pseudo-item $Y_{1i}$ for the first node is assigned value 0 if the observed response to item *i* falls into one of the disagreement categories 1, 2, 3 and value 1 if the response falls into one of the agreement categories 4, 5, 6. The related IRT model specifies the probability that person *v* shows an agreement response with $Y_{v1i}=1$ as a function of the trait θ, reflecting the assumption that the general (dis-)agreement decision is solely driven by the substantive trait, with item-specific discrimination parameter $\alpha ^{\text{ a }}_i$ and intercept parameter $d_{1i}$.

Pseudo-item $Y_{2i}$ for the second node has value 1 if the midscale category 3 is observed and value 0 if the non-midscale categories 1, 2 are observed. In contrast, pseudo-item $Y_{3i}$ for the third node has value 1 for the non-midscale categories 5, 6 and value 0 for midscale category 4, because all pseudo-items in Table 1 are coded in the direction of increasing response categories. Since pseudo-items $Y_{2i}$ and $Y_{3i}$ are defined conditional on a disagreement or agreement response, respectively, values are missing by design for $Y_{2i}$ and response categories 4, 5, 6 as well as for $Y_{3i}$ and categories 1, 2, 3. The IRT models for the second and third node in Table 1 contain the latent trait dimension θ together with dimension $\eta _\text {MRS}$ for individual differences in MRS, which mirrors the assumption that the selection of (non-)midscale categories on a rating scale is driven by both the substantive trait and midscale tendency. While the substantive trait θ is weighted by an item-specific discrimination parameter $\alpha ^{\text{ m }}_i$, the response style dimension $\eta _\text {MRS}$ is assumed to have constant influence over the rating items. Moreover, because the pseudo-item $Y_{3i}$ is coded in the direction of increasing categories and thus reversed with respect to the direction MRS, $\eta _\text {MRS}$ is included with a negative sign. As a consequence, larger values of $\eta _\text {MRS}$ increase the probability of midscale category 3 for disagreement responses, and they increase the probability of midscale category 4 for agreement responses by decreasing the probability of observing categories 5 and 6. Note that the sign reversal of the MRS dimension and the constant sign for the trait dimension between the pseudo-items $Y_{2i}$ and $Y_{3i}$ follow from coding all pseudo-items in the direction of increasing categories. An alternative coding scheme in the direction of response styles, where $Y_{3i}$ would be assigned value 1 for midscale category 4 and value 0 for the categories 5 and 6, would imply equal signs for $\eta _\text {MRS}$ in combination with a sign reversal for the substantive trait term $\alpha ^{\text{ m }}_i \, \theta$ (Meiser et al., 2019).

Finally, the pseudo-items $Y_{4i}$ and $Y_{5i}$ for the fourth and fifth node are conditionally defined for non-midscale responses of disagreement and agreement, respectively, and are missing by design for the remaining response categories. Pseudo-item $Y_{4i}$ distinguishes between the two leftmost response categories in the direction of ascending categories, with value 1 for the non-extreme category 2 and value 0 for extreme category 1. Analogously, pseudo-item $Y_{5i}$ distinguishes between the two rightmost response categories with value 0 for category 5 and value 1 for extreme category 6. The node-specific IRT models for $Y_{4i}$ and $Y_{5i}$ incorporate effects of the trait θ and of a response style dimension $\eta _\text {ERS}$, corresponding to the notion that the choice of a (non-)extreme response reflects joint effects of the substantive trait and of ERS. Like in the previous pseudo-items, the substantive trait is weighted with an item-specific discrimination parameter $\alpha ^{\text{ e }}_i$, whereas a constant impact over items is specified for the response style dimension. Due to the direction of coding, $\eta _\text {ERS}$ is included with a negative sign for $Y_{4i}$, so that a stronger ERS tendency increases the probability of observing extreme category 1 by decreasing the probability of category 2. Again, the sign reversal of the ERS dimension across the pseudo-items $Y_{4i}$ and $Y_{5i}$ is an implication of coding in the direction of increasing categories, and an alternative coding scheme in the direction of response styles, with $Y_{4i}$ taking value 1 for extreme rating category 1 and value 0 for rating category 2, would have resulted in a sign reversal for the substantive trait θ rather than for $\eta _\text {ERS}$.

Taken together, IRTree models conceptualize the original rating responses as combinations of pseudo-item responses along the branches of the tree, and the probability of an observed rating category follows from multiplying over the nodes of the branch that terminates in the given category (Böckenholt, 2012; Jeon & De Boeck, 2016). Because the observed response vectors are transformed into vectors of pseudo-item responses with an appropriate IRT parameterization, IRTree models can be specified and estimated in the broader framework of multidimensional IRT models using general-purpose IRT software like the R package mirt. The broader framework allows researchers to combine between-item and within-item multidimensionality as well as different response functions, like item-specific versus item-invariant loading parameters, in the node-specific IRT models. In the IRTree defined through Fig. 3 and Table 1, the specification of different loading parameters $\alpha ^\text {a}_i$, $\alpha ^\text {m}_i$ and $\alpha ^\text {e}_i$ for each rating item on the substantive trait θ permits varying strengths of trait effects from the overall (dis-)agreement decision in the top node to the fine-grained choices among the categories of gradual (dis-)agreement in the subsequent nodes of the IRTree. Moreover, while the node-specific models contain item-specific loading parameters for the substantive trait, the impact of the response style dimensions is constrained to be equal across items, paralleling the extended gPCM above. However, this restriction can be relaxed if items are assumed to be differentially susceptible to response style effects.

As with the extended IRT approach, it is straightforward to generalize the IRTree model in Fig. 3 and Table 1 to include more than one substantive trait for empirical applications with distinct subfactors or multiple personality variables. For that purpose, the weighted trait terms $\alpha ^{\text{ a }}_i \, \theta _{v}$, $\alpha ^{\text{ m }}_i \, \theta _v$ and $\alpha ^{\text{ e }}_i \, \theta _v$ in the right column of Table 1 can be replaced with sums $\sum _{h=1}^H \alpha ^{\text{ a }}_{hi} \, \theta _{hv}$, $\sum _{h=1}^H \alpha ^{\text{ m }}_{hi} \, \theta _{hv}$ and $\sum _{h=1}^H \alpha ^{\text{ e }}_{hi} \, \theta _{hv}$ over multiple traits $\theta _1,\ldots ,\theta _H$. As each rating item is assumed to refer to one substantive trait $\theta _h$, for each item *i* the loading parameters $\alpha ^{\text{ a }}_{hi}$, $\alpha ^{\text{ m }}_{hi}$ and $\alpha ^{\text{ e }}_{hi}$ are estimated for one latent dimension *h* and fixed to zero for all other dimensions (cf. Eq. 4). This generalization allows us to simultaneously analyze the two subfactors of metacognitive competence together with MRS and ERS in our empirical example.

### IRTree analysis of self-reported metacognitive competence

Next, we illustrate the implementation and estimation of the IRTree model in Fig. 3 for data on self-reported metacognitive competence (Double, 2025). For this purpose, we first have to compute the pseudo-items and specify the model structure with node-specific IRT models as outlined in Table 1. Then we can estimate the parameters of the IRTree as a multidimensional IRT model.

#### Definition of the pseudo-items and model structure

The decomposition of the rating responses with categories k=1,…,6 into binary pseudo-items according to Table 1 can be accomplished using standard R programming language, but an R package with special helper functions for IRTree analyses is also available (Li et al., 2026). With standard R language, the following code decomposes the original item responses into pseudo-items:^4^

**Figure:**
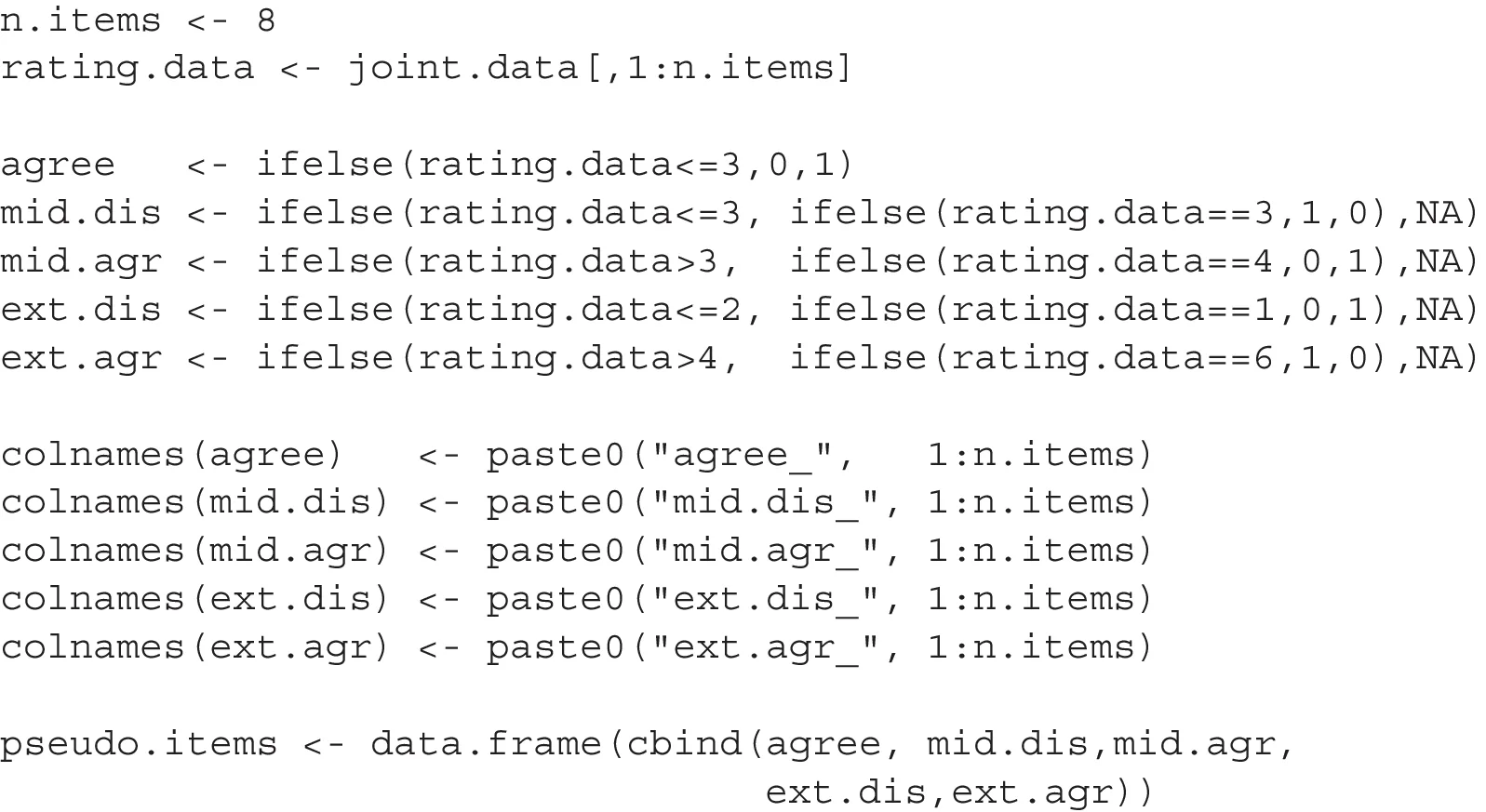

For each response of individual *v* to rating item *i*, the pseudo-item responses are computed for the agreement node (agree), the midscale node conditional on disagreement (mid.dis) or agreement (mid.agr), and the extremity node conditional on disagreement (ext.dis) or agreement (ext.agr). The values that are missing by design in Table 1 are defined as NA and thus treated as missing in the following analyses.

The resulting data.frame named pseudo.items contains the pseudo-item responses from the eight original rating items for each of the five nodes. Because the seventh rating item showed a zero frequency for the lowest category k=1, the corresponding pseudo-item for extremity given disagreement has a constant value 1. The pseudo-item ext.dis should therefore be removed for the seventh rating item to avoid estimation problems due to constant variable values. As the pseudo-item responses for extremity given disagreement are stored in columns 25,…,32 of the data.frame pseudo.items, we have to eliminate column 31 to drop the pseudo-item responses for the seventh rating item:

**Figure:**
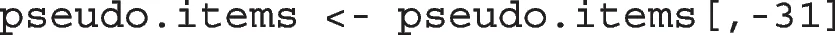

The reduced data.frame pseudo.items thus includes the pseudo-item responses from the eight rating items for the agreement node in columns 1–8, for the midscale nodes given disagreement or agreement in columns 9–16 and 17–24, and for the extremity nodes given disagreement or agreement in columns 25–31 and 32–39.

The pseudo-item responses form the data input for the IRTree analysis, which is further specified with the mirt.model function to define the latent structure of the model:

**Figure:**
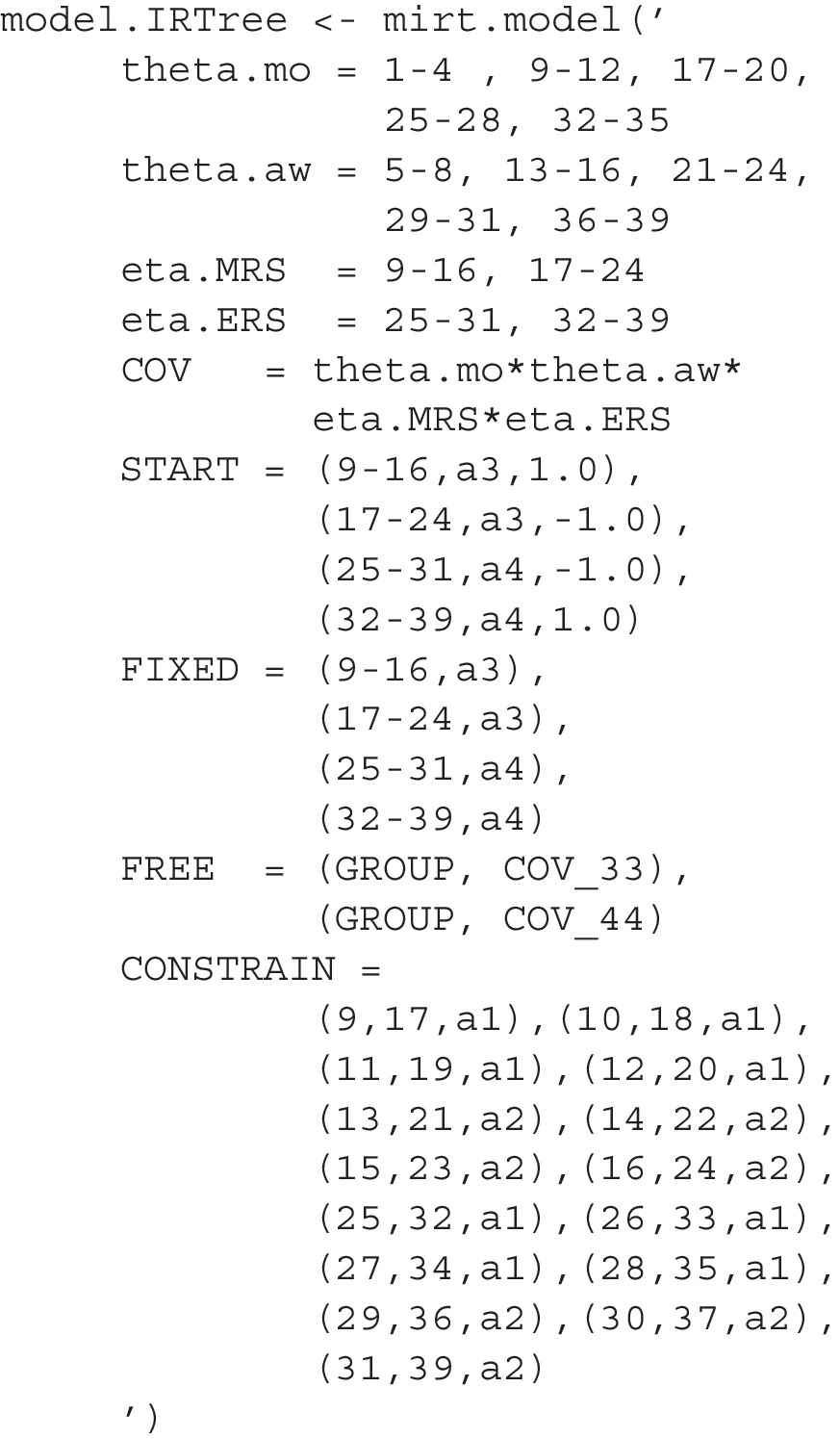

Here we define the two substantive traits theta.mo and theta.aw for “online monitoring” and “metacognitive awareness” together with the two response style dimensions eta.MRS and eta.ERS for MRS and ERS, respectively, and we assign the 39 pseudo-items in the data.frame pseudo.items to the latent factors. As the first four rating items refer to the first latent trait and the remaining four rating items refer to the second latent trait, the pseudo-item responses are split in quadruples between the factors theta.mo and theta.aw. In line with Table 1, the pseudo-items for the agreement node (i.e., columns 1–8 in pseudo.items) are solely assigned to one of the substantive traits, thereby generating unidimensional response functions, whereas the pseudo-items for the midscale nodes (i.e., columns 9–16 and 17–24 in pseudo.items) and extremity nodes (i.e., columns 25–31 and 32–39 in pseudo.items) are assigned to one substantive trait and one response style dimension each, thereby generating two-dimensional response functions. Like in the extended gPCM with response styles, a full covariance matrix of the four latent factors is included in the COV statement, which can easily be changed to impose orthogonality restrictions.

To keep the loadings of the response style dimensions at 1 or -1 despite estimating item-specific discrimination parameters for the two substantive trait dimensions, we use the START and FIXED commands in the mirt.model function. With the START option, we set the starting values for the loadings on $\eta _{\text{ MRS }}$ in the midscale-disagreement node to 1 and in the midscale-agreement node to -1. Likewise, we set the starting values for the loadings on $\eta _{\text{ ERS }}$ in the extremity-disagreement node to -1 and in the extremity-agreement node to 1. The FIXED command restricts the parameters to their designated starting values. Because the loading parameters on the response style dimensions are thus fixed at 1 or -1, the variances of the factors for MRS and ERS can be estimated as free parameters. We therefore overwrite the default restriction of fixed latent variances, which generally applies in mirt for models with item-specific discrimination parameters, for the two response style dimensions through the command FREE = (GROUP, COV_33),(GROUP, COV_44). Note that the latent variances count as group parameters as opposed to item parameters in mirt because they characterize the person distribution.

Finally, the CONSTRAIN keyword is employed to set the item-specific discrimination parameters for the substantive traits equal across the disagreement and agreement branches of the IRTree, where a1 and a2 refer to the discrimination parameters for the first dimension theta.mo and the second dimension theta.aw. More specifically, the pairwise constraints ensure that the item-specific discrimination parameters $\alpha _i^m$ for the midscale nodes are equated for midscale given disagreement and midscale given agreement, and that the item-specific discrimination parameters $\alpha _i^e$ for the extremity nodes are equated for extremity given disagreement and extremity given agreement. The model structure in model.IRTree thereby translates the node-specific IRT models in Table 1 into a joint multidimensional IRT framework for the 39 pseudo-item responses.

#### Estimation of the IRTree as a multidimensional IRT model

With the pseudo-item responses stored in the data.frame pseudo-items and with the model structure defined in the mirt.model model.IRTree, we can apply the IRTree model in Fig. 3 and Table 1 to the self-report data on metacognitive competence using the command mirt:

**Figure:**
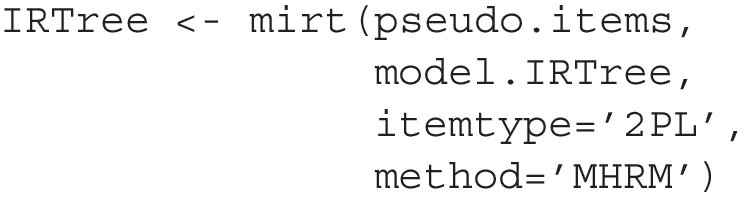

The mirt package takes a full information approach to parameter estimation, so that all existing pseudo-item responses are used, whereas the structurally missing values in pseudo.items are ignored without causing problems. Like in the extended gPCM model, we choose method=’MHRM’ to estimate the model with four latent dimensions. However, we specify itemtype=’2PL’ instead of itemtype=’gpcm’ to allow for item-specific discrimination parameters in the case of the binary pseudo-items.

With the coef command, we can display the estimated and fixed item parameters for the pseudo-items as well as the parameters of the multivariate person distribution for the two traits and two response styles:

**Figure:**
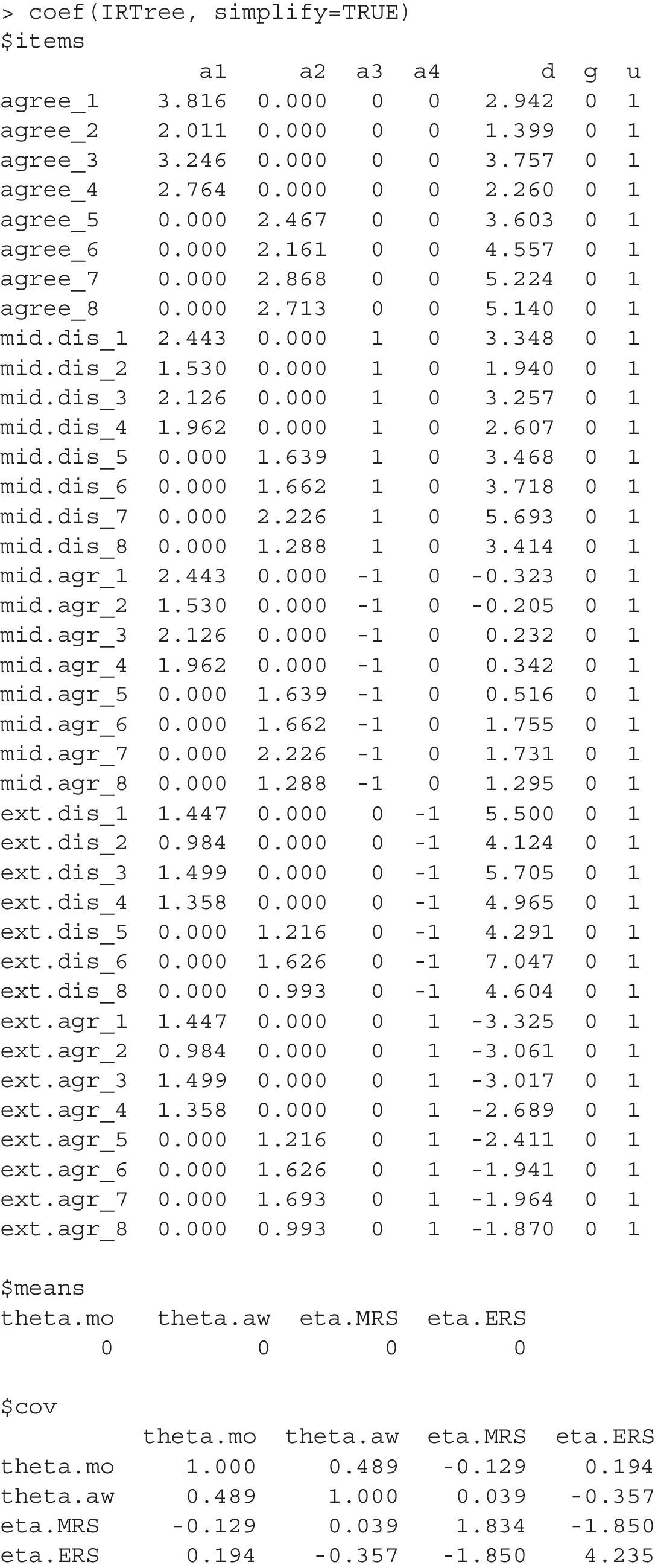

Starting with the item parameters, the columns a1 and a2 show the estimates of the discrimination parameters for the two substantive traits, that is, the parameters $\alpha _i^a$ for the pseudo-items of the agreement node, the parameters $\alpha _i^m$ for the pseudo-items of the midscale node conditional on disagreement and agreement, and the parameters $\alpha _i^e$ for the pseudo-items of the extreme node conditional on disagreement and agreement. The fixed zero values indicate the selective assignment of pseudo-items to one of the two traits and can be used to check whether the model was specified correctly. In addition, the estimated parameter values reflect the equality constraints for pseudo-items pertaining to the midscale and extreme nodes across disagreement and agreement responses (e.g., a1 for mid.dis_1 and mid.agr_1 or mid.dis_2 and mid.agr_2; and a2 for mid.dis_5 and mid.agr_5 or mid.dis_6 and mid.agr_6). A closer look at the estimates also shows that the discrimination parameters decrease for each of the two traits from the agreement node to the midscale and extremity nodes: Replicating earlier findings (Debelak et al., 2025; Meiser et al., 2019), the mean loadings on the trait of online monitoring in column a1 are largest for the agreement node with $\bar{\alpha }_{\cdot }^a=2.959,\,{ followedbythemidscalenodeswith}\bar{\alpha }_{\cdot }^m=2.015{ andtheextremenodeswith}\bar{\alpha }_{\cdot }^e=1.322$. A highly similar picture emerges for the loadings on the trait of metacognitive awareness in column a2 with $\bar{\alpha }_{\cdot }^a=2.552,\,\bar{\alpha }_{\cdot }^m=1.704{ and}\bar{\alpha }_{\cdot }^e= 1.382$.

Under the assumption of conditional independence over the nodes of the IRTree, these results support the notion that the substantive traits have a stronger influence on the overall (dis-)agreement decision than on the gradual choices among various response options of disagreement and agreement (Meiser et al., 2019). Similar results can emerge, however, if the node outcomes are conditionally dependent due to item-specific factors (Lyu et al., 2023), so that the differences in loading parameters across IRTree nodes should be interpreted with caution (see also Meiser and Reiber, 2023, for a discussion).

The next two columns, labeled a3 and a4, display the factor loadings on the two response style dimensions. The loadings for the MRS dimension in column a3 are fixed at zero for the pseudo-items of the agreement and extremity nodes and are set to 1 or -1 for the pseudo-items of the midscale node given disagreement and agreement. Analogously, the loadings for the ERS dimension in column a4 are fixed at zero for the pseudo-items of the agreement and midscale nodes but are set to $-1{ and}1$ for the pseudo-items of the extreme node given disagreement or agreement.

The column d provides the estimates of the intercept parameters for the pseudo-items (i.e., parameters $$d_{1i} { to}d_{5i} $$ in Table 1). Like in the extended gPCM, no constraints were imposed on the intercept parameters in the IRTree model. The intercept parameter of a pseudo-item indicates the overall tendency of the pseudo-item to show value 0 in the case of a negative intercept, and to show value 1 in the case of a positive intercept, assuming average person values on all latent dimensions. The rightmost two columns g and u contain guessing and slipping parameters that are irrelevant for the given IRTree analysis and remain fixed at 0 and 1, respectively.

Looking at the parameters of the multivariate person distribution, the latent expectations are restricted to zero by default (see section $ means in the output). In the latent covariance matrix (see section $ cov) the variances of the two substantive traits are restricted to 1, mirroring the default setting in mirt. The variances of the two response style dimensions are estimated freely, however, because we overwrote the default setting and released the restriction for the MRS and ERS dimensions in the model structure model.IRTree. The variance of the ERS dimension turns out to be larger than that of the MRS dimension, matching the results from the gPCM analysis above^5^ and earlier findings (Böckenholt & Meiser, 2017; Henninger & Meiser, 2020b), but the actual impact on response frequencies and bias depends on additional features like item difficulties relative to the person distribution (Schoenmakers et al., 2024). To inspect the linear relationships between the four latent dimensions, we can transform the latent covariance matrix into a correlation matrix:

**Figure:**
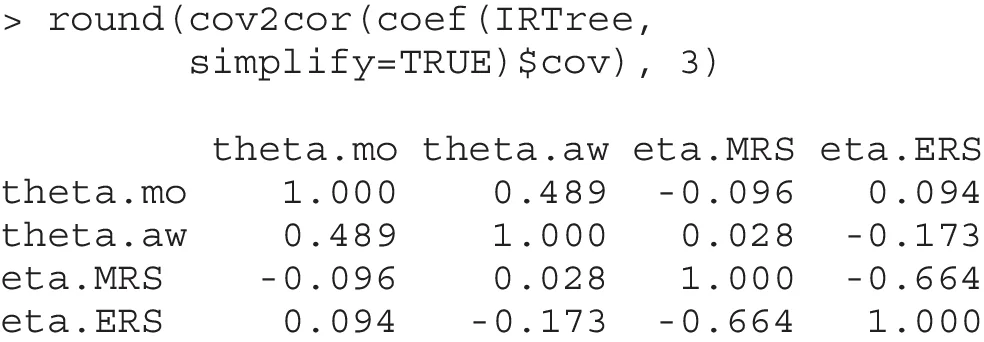

Resembling the results of the gPCM analysis, we find a pronounced positive correlation between the two substantive traits of online monitoring and metacognitive awareness, a strong negative correlation between the two response style dimensions of MRS and ERS, but only minor relationships between the substantive traits on the one hand and response styles on the other. We may thus conclude that the IRTree model successfully separates the substantive traits from response styles.

### Adjusting person estimates for response style effects in the IRTree model

**Fig. 4:**
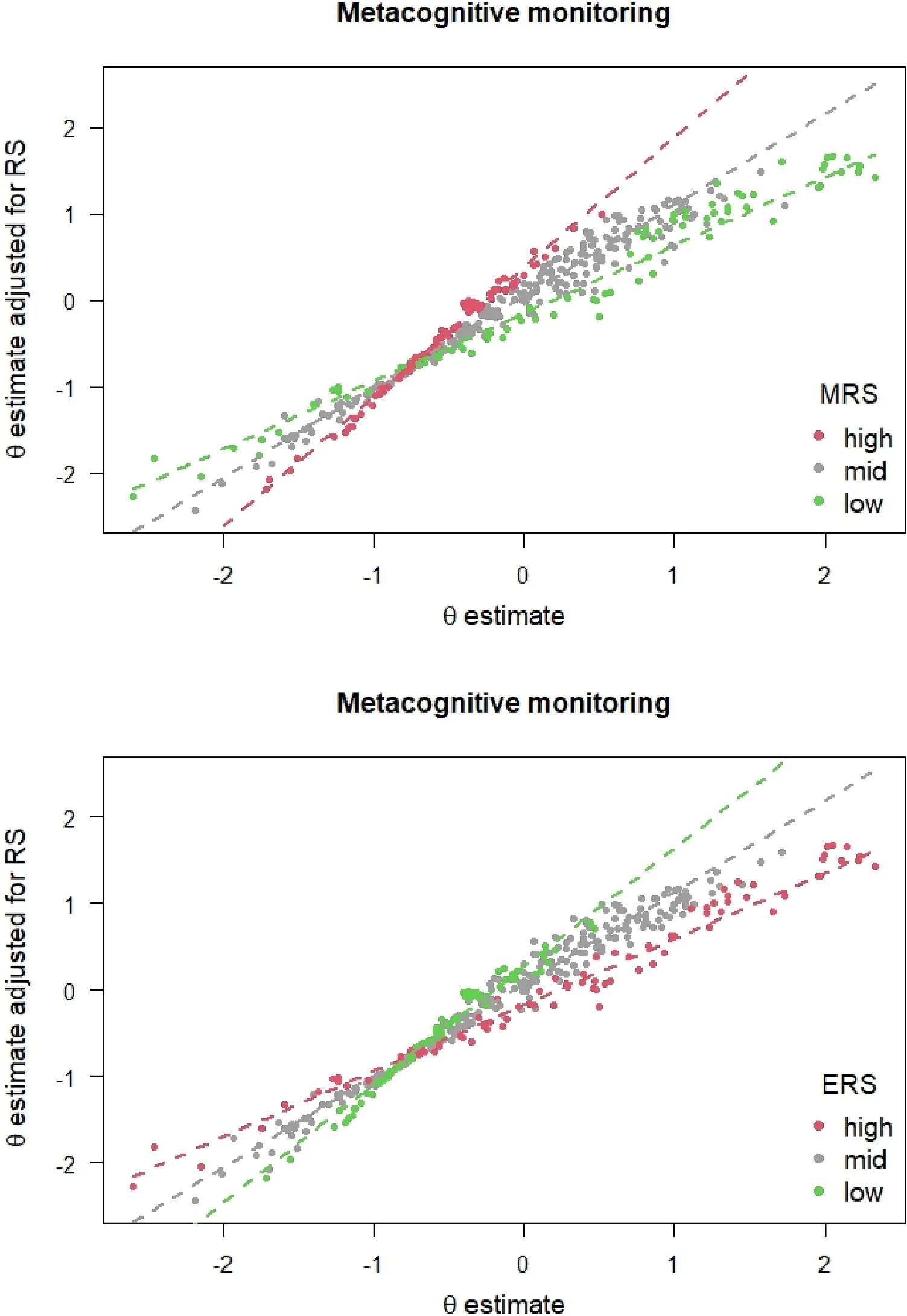
Trait estimates of metacognitive monitoring in the gPCM (*horizontal axis*) and in the IRTree model (*vertical axis*) for different levels of MRS and ERS

As with the extended IRT approach, IRTree models can adjust substantive trait estimates for individual differences in response styles. Figure 4 illustrates the model-implied adjustment through the IRTree model for the dimension of metacognitive monitoring and different levels of MRS (upper panel) and ERS (lower panel). The figure displays the trait estimates from the gPCM without response style factors on the horizontal axis as a baseline. The vertical axis shows the trait estimates from the IRTree model that accounts for MRS and ERS. As was the case in the extended IRT model (see Fig. 2), estimates from the IRTree model are amplified in either direction for high levels of MRS and low levels of ERS, but shrunk towards the mean for low levels of MRS and high levels of ERS. Due to the relative easiness of the given items compared to the sample of respondents, the adjustment is particularly evident for positive trait values with high levels of MRS and high levels of ERS (see Fig. 4).

## Discussion

Response styles reflect individual differences in response format use, regardless of item content. As such, they can lead to biases in the measurement of latent constructs and in the relations between measured constructs and other variables. In this tutorial, we have demonstrated how researchers can specify measurement models that accommodate response style factors to analyze the latent processes underlying observed rating responses and to adjust trait estimates for potential biases. The empirical application to self-reported metacognitive competence highlighted that response styles like MRS and ERS not only affect common assessments in personality research, but that they are also relevant for self-reported cognitive variables.

In our analyses, we outlined and illustrated two statistical approaches to control for response styles that follow different psychometric traditions, namely extended IRT models for ordinal data and multiprocess IRTree models. The first approach builds on partial credit and generalized partial credit models as established IRT frameworks for ordinal rating responses, and it conceptualizes response styles in terms of additional latent factors. The response style factors can increase or decrease the probabilities of certain response categories, like midscale or extreme categories, relative to the probabilities that are predicted for an individual’s trait value. The changes in category probabilities and the concomitant variations of the intervals between adjacent category thresholds on the latent continuum (see Fig. 1) bear a direct resemblance to the theoretical notion of response styles (Bolt et al., 2014; Falk & Cai, 2016; Henninger & Meiser, 2020a).

The second approach in terms of IRTree models rests on the theory-driven decomposition of observed rating responses into a series of intermediate decisions that form the nodes of a decision tree. The branches of the tree represent combinations of assumed decision processes which manifest in an observed response category, similar to multinomial processing models in experimental cognitive psychology (Schmidt et al., 2025). The decision tree for ordinal rating responses captures various stages of response selection, like overall (dis-)agreement, selection of a midscale response category and selection of an extreme category (see Fig. 3). Node-specific IRT models are specified to disentangle the underlying response processes along the tree, including processes based on the substantive trait and processes based on response styles (Böckenholt, 2012; Böckenholt & Meiser, 2017; Jeon & De Boeck, 2016; Meiser et al., 2019).

Despite the distinct traditions and rationales of extended IRT models for ordinal data on the one hand and IRTree models on the other, our application to the item responses on metacognitive competence (Double, 2025) yielded similar results for both statistical approaches. Either approach showed sizeable influences of the response style factors for MRS and ERS, with ERS consistently showing larger effects than MRS on the observed item responses. Moreover, both approaches proved successful in separating the substantive traits and the response style factors, as indicated by pronounced correlations among the substantive traits and among the response style factors but notably smaller correlations between traits and response styles. Over and above this correspondence of main findings, a direct comparison of the model-based estimates for the traits and response styles brings further evidence on the similarity of results: The trait estimates from the extended gPCM and those from the IRTree model revealed correlations of r=.997 for “online monitoring” and r=.993 for “metacognitive awareness”, and the estimates of the response style factors yielded correlations of r=.958 for MRS and r=.990 for ERS. Because psychometric modeling primarily aims to measure substantive traits, whereas response style factors statistically control potential biases, the nearly perfect correlations between the adjusted trait estimates suggest that the approaches are largely interchangeable for the given data. This similarity mirrors the parallel model definitions for the extended gPCM and IRTree models in the present analysis, which include multidimensional parameterizations for midscale and extreme responses, item-specific discrimination parameters for the substantive traits with constant discrimination parameters for the response style factors, and unconstrained intercept parameters for both modeling approaches. Because of the flexibility of both model classes, however, extended IRT and IRTree models can be specified with different features, which may lead to more divergent results from the two approaches to accounting for response styles.

Although this tutorial focused on the practical implementation and interpretation of multidimensional IRT and IRTree models to accommodate response styles, and although the results are similar, one should not overlook the conceptual differences when choosing a modeling framework for one’s research. Whereas the latent dimensions are selectively assigned to the individual nodes for the response categories in the IRTree framework (see Table 1), all latent dimensions enter the model equation for each of the categories in the extended IRT framework through the common denominator in divide-by-total models (see Eqs. 3 and 4). As a consequence, in extended IRT models, response styles like extreme responding affect not only the choices between extreme and non-extreme categories of (dis-)agreement but can also affect the probability of overall agreement with an item, which is not the case for IRTree models (Schoenmakers et al., 2024). On the other hand, conditional on a (non-)extreme response, the probability of choosing (non-)extreme agreement rather than disagreement may vary not only as a function of the substantive trait but also of the level of ERS in an IRTree if item difficulty mismatches the sample of respondents, which is not observed in an extended IRT model (Schoenmakers et al., 2024). Beyond these implications of different model definitions, choosing a modeling framework entails assuming that the model captures the true data-generating process, and misspecification can bias structural model parameters and lead to under- or overadjustment of trait estimates. Researchers must therefore decide, on theoretical grounds, which model best fits their data and research question.

While model choice is relevant when accounting for response styles, choosing a model that accommodates response styles is generally better than not accommodating response styles at all if individual differences in response styles are present in the data (Falk & Cai, 2016; Schoenmakers et al., 2024). In the absence of theoretical reasons to favor one model class over the other, applied researchers may therefore draw on pragmatic considerations and take the complexity of model specification into account. If their primary goal is to adjust trait estimates for potential biases, extended IRT models may be the natural choice because they do not require the definition of pseudo-items and can be implemented straightforwardly and with concise R code. If, on the other hand, the main objective is the detailed analysis of latent processes driving observed categorical responses, IRTree models may be the preferred option because they allow researchers to explicitly specify and test assumptions about contingent latent response processes for individual categories through the theory-driven definition and parameterization of pseudo-items.

Previous comparisons of psychometric models for response styles examined IRT models for discrete response style dimensions together with IRTree models for continuous response styles (Böckenholt & Meiser, 2017), focused selectively on the class of extended IRT models for ordinal data (Falk & Ju, 2020; Henninger & Meiser, 2020a, b), analyzed IRT and IRTree models with a particular focus on multi-group designs (Schoenmakers et al., 2024) , or offered a theoretical overview of the assumptions and consequences that are associated with the different statistical approaches (Bolt & Meng, 2025). In contrast, this tutorial provides a practical guide to using state-of-the-art IRT and IRTree models for continuous response style factors, with elements from recent psychometric developments. In particular, we showed how extended IRT models for ordinal data and IRTree models can be implemented with standard R tools, and how the parameters can be estimated in the common framework of multidimensional IRT modeling using the R package mirt (Chalmers, 2012; Chalmers et al., 2025). Deviating from traditional IRTree specifications with unidimensional node parameterizations (Böckenholt, 2012; Böckenholt & Meiser, 2017; Jeon & De Boeck, 2019; Plieninger & Meiser, 2014), we built on recent generalizations to multidimensional node parameterizations which allow researchers to differentiate between trait and response style effects in the selection of midscale and extreme responses (Meiser et al., 2019; Schoenmakers et al., 2024).

Simulation studies showed that IRTree models with unidimensional node parameterizations tend to misattribute true trait variance to response-style dimensions when items are overall too difficult or too easy for the person sample, whereas IRTree models with multidimensional node parameterizations are more robust to such mismatches (Merhof et al., 2024). It is therefore recommended to specify multidimensional node models if theoretical considerations suggest that traits and response styles are jointly involved in a decision along the IRTree. Importantly, for the metacognitive data used herein, the multidimensional node parameterization ensured low correlations between substantive trait dimensions and response style factors despite skewed response frequencies, which indicates successful separation of response processes (see Merhof et al., 2024, for details). Last but not least, we demonstrated the flexibility to combine item-specific and item-invariant discrimination parameters for different latent dimensions of an IRTree, while previous model definitions were mostly restricted to item-invariant discrimination for all factors (Debelak et al., 2025; Meiser et al., 2019).

In our empirical application, we illustrated the statistical control of MRS and ERS in responses to items with a six-point rating scale. While MRS and ERS exert symmetrical effects in both directions to or from the midpoint of the rating scale, the analysis of response styles can be generalized to non-symmetrical response styles (e.g., Adams et al., 2019; Henninger, 2021). A prominent case of non-symmetrical response style effects comes from acquiescence, that is, the tendency to agree irrespective of item content (Baumgartner & Steenkamp, 2001). In fact, extended IRT models for ordinal data and IRTree models have also been proposed and used to account for acquiescence (Plieninger & Heck, 2018; Wetzel & Carstensen, 2017), but the analysis of acquiescent response style requires reversed items that were not included in the item set on self-reported metacognitive competence (Double, 2025). If reversed items are available, an additional acquiescence factor can be included through appropriate scoring weights in an extended IRT model for ordinal data and through the tailored parameterization of node probabilities in an IRTree model. Likewise, IRT and IRTree models for response styles can easily be constructed for different numbers of response categories by adapting the scoring weight matrices or modifying the definition of pseudo-items according to the response format.

The extended IRT and IRTree approaches that were illustrated and used herein have been introduced in the previous literature together with their identifiability conditions, like fixed expectations of latent dimensions to determine the scale origins and fixed latent variances to determine the metrics of measurement where appropriate. Simulation studies confirmed good estimation properties of the structural and person parameters in either approach, including small bias and high recovery, over a range of sample sizes and test lengths (e.g., Alagöz et al., 2025; Falk & Cai, 2016; Henninger & Meiser, 2020a). Nevertheless, some design features can help to disentangle latent response processes in multidimensional IRT and IRTree models. For example, modeling more than one substantive trait facilitates separating trait dimensions and response-style factors that are invariant across trait domains, especially when the traits are only moderately correlated. Moreover, item difficulties or thresholds should be dispersed over an appreciable range, and mean item difficulty should not severely mismatch the person distribution in the sample, to efficiently distinguish trait and response style effects (e.g., Henninger & Meiser, 2022; Merhof et al., 2024; Schoenmakers et al., 2024; Wetzel & Carstensen, 2017). Beyond study design, researchers using multidimensional IRT and IRTree models for response styles can inspect standard errors to detect potential threats to estimation precision or use these models as tools of sensitivity analyses for their research goals (Bolt & Meng, 2025).

A common caveat to the presented IRT and IRTree models for response styles concerns the inherent homogeneity assumption of response processes over items and respondents. While the models separate response processes that are due to individual differences in substantive traits and response styles into different latent dimensions, the models maintain the premise that these latent dimensions influence the observed responses to the same degree for all items and for all individuals. Recent developments in IRTree modeling, however, allow for systematic variability of trait and response style effects as a function of item position (Merhof & Meiser, 2023) and thus release the homogeneity assumption across items. An empirical application showed that the impact of response styles relative to trait effects increased over the course of a longer test, suggesting a change from effortful to more heuristic response behavior. In a similar vein, IRTree models for response styles were combined with discrete mixture distributions (Alagöz & Meiser, 2024; Alagöz et al., 2025; Kim & Bolt, 2021) and score-based partitioning methods (Debelak et al., 2025) to relax the homogeneity assumption across respondents, and empirical analyses corroborated the existence of subpopulations with different weightings of the trait and response style dimensions. These recent developments enable researchers to investigate sources of heterogeneity in latent response processes, and they increase the flexibility to use IRTree models for the tailored measurement of latent traits adjusted for individual response strategies.

Finally, it is worth noting that the models discussed in this tutorial address response styles in the context of Likert-type scales, which yield ordinal rating responses. While such formats remain by far the most common in psychological research and related disciplines, visual analog scales, in which respondents indicate their level of agreement by positioning a continuous slider, have gained considerable traction in recent years. Readers interested in modeling response styles for the resulting continuous bounded responses are referred to Jiang et al. (2025) and Vollbracht et al. (2026) for recent developments in this area.

To conclude, IRT models for ordinal data and IRTree models offer a rich toolbox for accounting for response styles when measuring latent constructs, in combination with facilitatory questionnaire design. In this tutorial, we provided an introduction to IRT and IRTree models for response styles with a practical guide to model specification and estimation. As becomes obvious from the foregoing discussion of recent developments in IRTree modeling, the control of response styles is an ongoing field of psychometric research that has implications for statistical innovation as well as for applied assessment in various fields of psychology and education.

## Data Availability

The data used for illustration were taken from Double 2025 and can be retrieved at https://osf.io/4jyxb/files/osfstorage

## Footnotes

1 Note that for customary IRT models with within-item multidimensionality the usual interpretation of threshold parameters in the PCM or gPCM would not be tenable: The threshold parameters $\beta _{ij}$ in Eqs. 1 and 2 can be interpreted as the difficulty to select category *j* over category j-1 given that the response is either j-1 or *j* (Masters, 1982; Muraki, 1992), where the difficulty refers to the trait dimension θ. Because difficulty parameters usually cannot be uniquely assigned to a specific dimension in models with more than one person dimension in the item response function, the notation is adjusted to that of confirmatory factor models with factor loading $\alpha _i$ and intercept parameter $d_{ik}$.

2 The data from Double 2025 can be downloaded from the OSF project of the original article at https://osf.io/4jyxb/files/osfstorage. The data from the first study are also available in the Item Response Warehouse (IRW) collection (Domingue et al., 2025).

3 The baseline gPCM with two dimensions can be conceived of as a special case of the extended gPCM with four dimensions that follows from the restrictions $\sigma ^2_{\eta _{\text {MRS}}} = \sigma ^2_{\eta _{\text {ERS}}} = 0$ at the boundary of the parameter space. The true reference distribution of the likelihood ratio test statistic is therefore a mixture of chi-square distributions (Stoel et al., 2006), and the use of the non-mixture chi-square distribution with 7 degrees of freedom in the anova output implies a too conservative test for this situation. As a consequence, the significant result can be interpreted without risking an artifact, whereas a non-significant outcome would potentially have been due to a lack of power (Hoffman, 2015).

4 The R package irtrees (Li et al., 2026) contains the function dendrify that decomposes a matrix of item responses into pseudo-item responses in long data format according to a user-designed mapping matrix. Researchers who find the helper function more convenient than standard R syntax can define the mapping matrix for the IRTree in Fig. 3 and Table 1 as mapping <- cbind(c(0,0,0,1,1,1), c(0,0,1,NA,NA,NA), c(NA,NA,NA,0,1,1), c(0,1,NA,NA,NA,NA), c(NA,NA,NA,NA,0,1)). The transformation to long data format with the function dendrify is especially suitable if IRTrees are estimated in the framework of generalized linear mixed models (De Boeck & Partchev, 2012). The standard R code used in this tutorial transforms the item responses into pseudo-items in a wide data format that can directly be transmitted to mirt and is therefore preferred here.

5 Note that the stronger effect of ERS relative to MRS is observed in terms of a larger variance in the IRTree analysis in which the factor loadings are fixed, but in terms of larger factor loadings in the gPCM analysis in which the latent variances are fixed. This difference is merely due to the varying scaling restrictions, which are commonly imposed on the latent variances but can be shifted to the loadings for convenience, and it does not bear any conceptual implications.

## Declarations

### Code availability

The R script for this tutorial is available on the OSF and can be retrieved via the following link: https://osf.io/cbwun/files/osfstorage
